# Supplementary material for: Identification of Freezing-Responsive microRNAs and Their Targets in Chinese Jujube by Small RNA and Degradome Sequencing
Source: Int J Mol Sci. 2026 Sep 4;27(17):7912. doi: 10.3390/ijms27177912 (PMC13566890; doi:10.3390/ijms27177912)
Supplement: Supplementary file 1 [file ijms-27-07912-s001.zip › Supplementary Material S2/novel_miR_161.pdf]

Provisional ID	: novel_miR_161
Score total	: 229578.7
Score for star read(s)	: 3.9
Score for read counts	: 229571.8
Score for mfe	: 1.4
Score for randfold	: 1.6
Score for cons. seed	:
Total read count	: 450307
Mature read count	: 395695
Loop read count	: 20
Star read count	: 54592

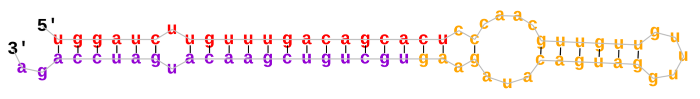

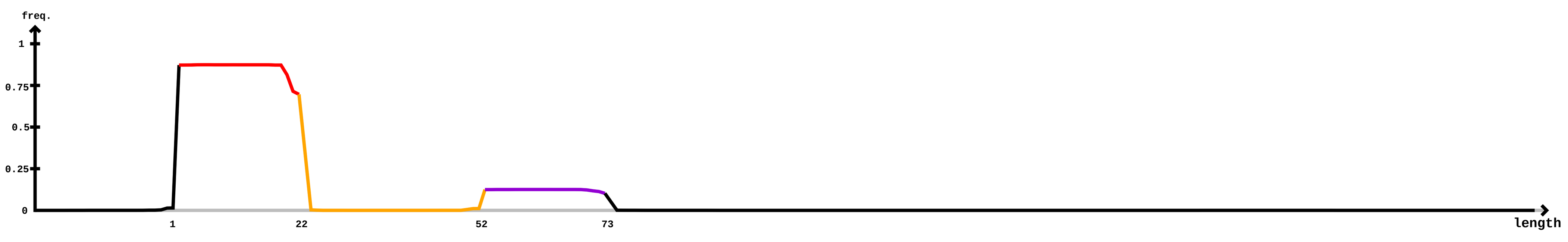

Mature Star

```
5'- cgcauguugcucguuuaccuucuggaucuuguuugacagcacucccaacguuguuguuuggaugacauagaagugcugucgaacaugauccagaaggugcugucgaacaacauacaaaaaggguuuacuuuacguaccgcccaucacgaucuucuucuucuucuucuuccugaaagaguaucaaacaugggguuucuguuuguuucucuuuuucuuuuuuaaaguagauuuacauguuaacuguauugc -3' obs
    cgcauguugcucguuuaccuucuggaucuuguuugacagcacucccaacguuguuguuuggaugacauagaagugcugucgaacaugauccagaaggugcugucgaacaacauacaaaaaggguuuacuuuacguaccgcccaucacgaucuucuucuucuucuucuuccugaaagaguaucaaacaugggguuucuguuuguuucucuuuuucuuuuuuaaaguagauuuacauguuaacuguauugc     exp
    ...(((((.(((..((((((((((((.(((((((((((((.(....((((((.....))))))...).)))))))))))))).)))))))))))))....))).))))))........((((.(((.....)))..))))............(((.(((.........(((((((...((((((.((....))))))))...))))))).........))).)))......................
```

	reads	mm	sample
..cauguugUucguuuaccuuc..	2	1	LT-3
..cauguugcucguuuaccuucuggaucuugA..	1	1	JCK-3
..cauguugcucguuuaccuucuggaucuugA..	1	1	JCK-2
...auguugUucguuuaccuucug..	2	1	JCK-2
...auguugUucguuuaccuucug..	3	1	JCK-1
....uguugcucguuuaccuucug..	1	0	LT-1
....uguugUucguuuaccuucugg..	2	1	JCK-1
....uguugcucguuuaccuucugg..	10	0	JT-2
....uguugUucguuuaccuucugg..	2	1	JCK-2
....uguugcucguuuaccuucugg..	7	0	JT-3
......uugUucguuuaccuucuggauc..	2	1	JCK-1
......uugUucguuuaccuucuggauc..	6	1	JCK-2
......uugUucguuuaccuucuggauc..	2	1	LT-2
......uugUucguuuaccuucuggauc..	3	1	JCK-3
.......ugUucguuuaccuucuggauc..	12	1	LT-3
.......ugUucguuuaccuucuggauc..	30	1	JCK-3
.......ugUucguuuaccuucuggauc..	10	1	JCK-1
.......ugUucguuuaccuucuggauc..	31	1	JCK-2
.......ugUucguuuaccuucuggauc..	12	1	LT-1
.......ugUucguuuaccuucuggauc..	3	1	LT-2
.........cucguuuaccuucuggaucuuguu..	1	0	LT-1
..........ucguuuaccuucuggaucuug..	6	0	LCK-3
..............uuaccuucuggaucuuguuug..	2	0	JCK-2
................accuucuggaucuuguuug..	2	0	LCK-3
................accuucuggaucuuguuuga..	1	0	JCK-2
................accuucuggaucuuguuuga..	2	0	LT-3
................accuucuggaucuuguuuga..	4	0	LT-2
................acNuucuggaucuuguuuga..	1	1	LT-2
................accuucuggaucuuguuuga..	3	0	LT-1
................accuucuggaucuuguuuga..	2	0	JCK-1
................accuucuggaucuuguuugac..	2	0	LT-3
................accuucuggaucuuguuugaA..	1	1	JCK-1
................accuucuggaucuuguuugac..	1	0	JCK-2

Mature Star

cgcauguugcucguuuaccuucuggaucuuguuugacagcacucccaacguuguuguuuggaugacauagaagugcugucgaacaugauccagaaggugcugucgaacaacauacaaaaagggguuuacuuuacguaccgcccaucacgaucuucuucuucuucuucuuccugaaagaguaucaaacaugggguuucuguuuguuucucuuuuucuuuuuuaaaguagauuuacauguuaacuguauugc

................accuucuggaucuuguuugac	4	0	LT-1
................accuucuggaucuuguuugac	3	0	JCK-3
................accuucuggaucuuguuugaca	2	0	JCK-1
................accuucuggaucuuguuugacagc	1	0	JCK-3
................accuucuggaucuuguuugacagc	1	0	JCK-1
................accuucuggaucuuguuugacagc	2	0	JCK-2
.................ccuucuggaucuuguuuga	4	0	LT-2
.................ccuucuggaucuuguuuga	6	0	LCK-3
.................ccuucuggaucuuguuugac	1	0	LT-1
.................ccuucuggaucuuguuugac	7	0	LT-2
.................ccuucuggaucuuguuugaca	25	0	LT-2
.................ccuucuggaucuuguuugaca	27	0	LT-3
.................cNuucuggaucuuguuugaca	1	1	JCK-1
.................ccuucuggaucuuguuugaca	46	0	JCK-2
.................ccuucuggaucuuguuugaca	37	0	JT-1
.................ccuucuggaucuuguuugaca	15	0	LT-1
.................ccuucuggaucuuguuugaca	33	0	JCK-3
.................ccuucuggaucuuguuugaca	34	0	LCK-1
.................ccuucuggaucuuguuugCca	1	1	LCK-2
.................ccuucuggaucuuguuugaca	31	0	JCK-1
.................ccuucuggaucuuguuugaca	37	0	LCK-3
.................ccuucuggaucuuguuugaca	18	0	LCK-2
.................ccuucuggaucuuguuugacagc	3	0	JCK-3
.................ccuucuggaucuuguuugacagc	2	0	LT-1
.................ccuucuggaucuuguuugacagcac	1	0	LT-1
.................ccuucuggaucuuguuugacagcacuccc	3	0	LCK-3
..................cuucuggaucuuguuugaca	4	0	JCK-2
..................cuucuggaucuuguuugaca	2	0	LT-3
..................cuucuggaucuuguuugacU	1	1	LT-3
..................cuucuggaucuuguuugaca	1	0	JCK-1
..................cuucuggaucuuguuugaca	8	0	LCK-3
..................cuucuggaucuuguuugaca	5	0	LT-2
..................cuucuggaucuuguuugaAa	1	1	JCK-2
..................cuucuggaucuuguuugaca	2	0	JCK-3
..................cuucuggaucuuguuugacagc	2	0	LT-3
..................cuucuggaucuuguuugacagc	1	0	JCK-3
..................cuucuggaucuuguuugacagc	2	0	JCK-1
..................cuucuggaucuuguuugacagc	4	0	JCK-2
..................cuucuggaucuuguuugacagca	2	0	JCK-1
..................cuucuggaucuuguuugacagca	1	0	JCK-3
..................cuucuggaucuuguuugacagca	1	0	JCK-2
...................uucuggaucuuguuugac	6	0	LT-2
...................uucuggaucuuguuugaca	1	0	LT-2
...................uucuggaucuuguuugaca	1	0	JCK-1
...................uucuggaucuuguuugaca	11	0	JT-1
...................uucuggaucuuguuugaca	5	0	LT-3
...................uucuggaucuuguuugaca	3	0	JCK-3
...................uucuggaucuuguuugacU	9	1	JT-1
...................uucuggaucuuguuugaca	1	0	LCK-3
...................uucuggaucuuguuugacag	2	0	LT-2
...................uucuggaucuuguuugacaU	1	1	LT-3
...................uucuggaCcuuguuugacagc	1	1	LT-1
...................uucuggCucuuguuugacagc	1	1	LT-2
...................uucuggaucuuguuugacagc	43	0	LCK-1
...................uucuggaucuuguuugacagc	129	0	LT-1
...................uucugAaucuuguuugacagc	1	1	LT-1
...................uucuggaucuuguuugacagc	43	0	JCK-2
...................uucuggaucuuguuugacagc	105	0	LT-2
...................uucuggaucuuguuugacagN	1	1	JCK-1
...................uucuggaucuuguuugacagc	39	0	JCK-3
...................uucuggaucuugCuugacagc	1	1	LCK-3
...................uucuggCucuuguuugacagc	1	1	LT-1
...................uucuggaucuuguuugacagc	139	0	LT-3
...................uucuggaucuuguuugacagc	29	0	JCK-1
...................uucuggaucuuguuugacCgc	1	1	LT-1
...................uucuggaucuuguuugacagc	38	0	LCK-2
...................uucuggaucuuguuugacagc	131	0	LCK-3
...................uucuggaucuuguuugacagcU	3	1	LT-3
...................uucuggaucuuguuugacagcG	1	1	LT-3
...................uucuggaucuuguuugacagcU	7	1	LT-2

```
                    Mature                                    Star
cgcauguugcucguuuaccuucuggaucuuguuugacagcacucccaacguuguuguuuggaugacauagaagugcugucgaacaugauccagaaggugcugucgaacaacauacaaaaagggguuuacuuuacguaccgcccaucacgaucuucuucuucuucuucuuccugaaagaguaucaaacaugggguuucuguuuguuucucuuuucuuuuuuaaaguagauuuacauguuaacuguauugc
...................uucuggaucuuguuugacagca........................................................................................................................................................................................................    1     0     JCK-3
...................uucuggaucuuguuugacagcU........................................................................................................................................................................................................    4     1     LCK-3
...................uucuggaucuuguuugacagcU........................................................................................................................................................................................................    5     1     LT-1
...................uucuggaucuuguuugacagcacu.....................................................................................................................................................................................................    3     0     LCK-3
....................ucuggaucuuguuugaca...................................................................................................................................................................................................................    3     0     LCK-1
....................ucuggaucuuguuugaca...................................................................................................................................................................................................................    79    0     LCK-3
....................ucuggaucuuguuugacU...................................................................................................................................................................................................................    2     1     LT-3
....................ucuggaucuuguuugaca...................................................................................................................................................................................................................    3     0     LCK-2
....................ucuggaucuuguuugaca...................................................................................................................................................................................................................    28    0     LT-1
....................ucuggaucuuguuugaca...................................................................................................................................................................................................................    25    0     JCK-1
....................ucuggaucuuguuugaca...................................................................................................................................................................................................................    15    0     JCK-3
....................ucuggaucuuguuugacU...................................................................................................................................................................................................................    1     1     JCK-1
....................ucuggaucuuguuugaca...................................................................................................................................................................................................................    33    0     JCK-2
....................ucuggaucuuguuugaca...................................................................................................................................................................................................................    9     0     JT-1
....................ucuggaucuuguuugaca...................................................................................................................................................................................................................    35    0     JT-3
....................ucuggaucuuguuugaca...................................................................................................................................................................................................................    41    0     LT-2
....................ucuggaucuuguuugacU...................................................................................................................................................................................................................    1     1     JCK-2
....................ucuggaucuuguuugacU...................................................................................................................................................................................................................    6     1     LT-1
....................ucuggaucuuguuugacU...................................................................................................................................................................................................................    9     1     LCK-3
....................ucuggaucuuguuugaca...................................................................................................................................................................................................................    30    0     JT-2
....................ucuggaucuuguuugaca...................................................................................................................................................................................................................    26    0     LT-3
....................ucuggaucuuguuugacag..................................................................................................................................................................................................................    2     0     LT-1
....................ucuggCucuuguuugacagc.................................................................................................................................................................................................................    1     1     LCK-1
....................ucuggaucuuguuugacagc.................................................................................................................................................................................................................    29    0     JCK-1
....................ucuggaucuuguuugacagc.................................................................................................................................................................................................................    75    0     LCK-2
....................ucuggaucuuguuugacagc.................................................................................................................................................................................................................    41    0     LCK-3
....................ucuggaucuuguuuUacagc.................................................................................................................................................................................................................    1     1     LCK-2
....................ucuggaucuuguuugacagc.................................................................................................................................................................................................................    55    0     JCK-2
....................uGuggaucuuguuugacagc.................................................................................................................................................................................................................    1     1     LCK-1
....................ucuggaucuuguuugacaUc.................................................................................................................................................................................................................    1     1     LT-1
....................ucuggaucuuguuuNacagc.................................................................................................................................................................................................................    1     1     LCK-1
....................ucuggaucuuguuugacagA.................................................................................................................................................................................................................    1     1     JCK-1
....................ucuggaucuuguuugacagc.................................................................................................................................................................................................................    63    0     LCK-1
....................ucuggaucuuguuugacagc.................................................................................................................................................................................................................    127   0     LT-3
....................ucuggCucuuguuugacagc.................................................................................................................................................................................................................    2     1     LT-2
....................ucuggaucuuguuugacagc.................................................................................................................................................................................................................    116   0     LT-2
....................ucuggaucuuguuugacagc.................................................................................................................................................................................................................    35    0     JCK-3
....................ucuggaucuuguuugacagA.................................................................................................................................................................................................................    1     1     JCK-3
....................ucuggaucuuguuugacagA.................................................................................................................................................................................................................    1     1     LT-3
....................ucuggaucuuguuugacagc.................................................................................................................................................................................................................    107   0     LT-1
....................ucuggaucuuguuugacagca................................................................................................................................................................................................................    29    0     JT-1
....................ucuggaucuuguuugacagUa................................................................................................................................................................................................................    1     1     JCK-2
....................ucuggaucuuguuugCcagca................................................................................................................................................................................................................    1     1     LT-1
....................ucuggCucuuguuugacagca................................................................................................................................................................................................................    1     1     JCK-2
....................ucuggaucuuguuugacagca................................................................................................................................................................................................................    145   0     LCK-3
....................ucuggaucuuguuugacagca................................................................................................................................................................................................................    195   0     LT-2
....................ucuggaucuuguuugacagca................................................................................................................................................................................................................    1097  0     JCK-2
....................ucuggaucuuguuugacagca................................................................................................................................................................................................................    17    0     LCK-2
....................ucuggaucuuguuugacagcN................................................................................................................................................................................................................    1     1     JCK-1
....................uUuggaucuuguuugacagca................................................................................................................................................................................................................    8     1     JCK-3
....................ucuggaucuuguuugUcagca................................................................................................................................................................................................................    1     1     JCK-3
....................ucuggaucuuguuugacaUca................................................................................................................................................................................................................    3     1     JCK-3
....................ucuggaucuuguuugacagcC................................................................................................................................................................................................................    1     1     LCK-3
....................ucuggaucuuguuugacagca................................................................................................................................................................................................................    9     0     LCK-1
....................ucuggaucuCguuugacagca................................................................................................................................................................................................................    1     1     LT-3
....................ucuggaucuuguuugacagca................................................................................................................................................................................................................    299   0     LT-1
....................uNuggaucuuguuugacagca................................................................................................................................................................................................................    1     1     JCK-1
....................ucuggaucuuguuugacagca................................................................................................................................................................................................................    11    0     JT-2
....................ucCggaucuuguuugacagca................................................................................................................................................................................................................    1     1     JCK-2
....................ucuggauAuuguuugacagca................................................................................................................................................................................................................    1     1     JCK-2
....................ucuggaucuuguuugacagNa................................................................................................................................................................................................................    2     1     JCK-1
....................ucuggaucuugGuugacagca................................................................................................................................................................................................................    1     1     JCK-1
....................ucuggaucuuguuugacagcU................................................................................................................................................................................................................    3     1     JCK-3
....................ucuggauUuuguuugacagca................................................................................................................................................................................................................    1     1     JCK-2
....................ucuggaucuuguuugGcagca................................................................................................................................................................................................................    1     1     LT-2
....................ucuggaucuuguuugacagca................................................................................................................................................................................................................    267   0     LT-3
....................ucuggaucuugGuugacagca................................................................................................................................................................................................................    2     1     LT-1
....................ucuNgaucuuguuugacagca................................................................................................................................................................................................................    1     1     JCK-1
....................ucuggaucuuguuugCcagca................................................................................................................................................................................................................    2     1     LT-3
....................ucuggaucuuguuugacagca................................................................................................................................................................................................................    628   0     JCK-1
```

```
                 Mature                               Star
cgcauguugcucguuuaccuucuggaucuuguuugacagcacucccaacguuguuguuuggaugacauagaagugcugucgaacaugauccagaaggugcugucgaacaacauacaaaaaggggguuuacuuuacguaccgcccaucacgaucuucuucuucuucuucuuccugaaagaguaucaaacaugggguuucuguuuguuucucuuuuucuuuuuuaaaguagauuuacauguuaacuguauugc
....................ucuggaucuuguuugacagcU      6    1   JCK-1
....................ucuggaucuuguuugUcagca      1    1   JCK-2
....................ucuggaucuuguuugacagcU      3    1   JCK-2
....................ucuggaucuuguuugacaUca      5    1   JCK-2
....................ucCggaucuuguuugacagca      1    1   JCK-1
....................ucuggaucuuguuugacagcU      14   1   LT-2
....................ucuggaucuuguuugacagca      832  0   JCK-3
....................ucuggaucuuguuugacagcU      6    1   LT-1
....................ucuggaucuuguuugacagcU      7    1   LT-3
....................ucuggaucuuguuugacaUca      2    1   JCK-1
....................uUggaucuuguuugacagca       1    1   JCK-1
....................ucuggaucuuguuugacagcU      9    1   LCK-3
....................ucuggaucuuguuugacagca      12   0   JT-3
....................ucuggaucuuguuugGcagca      1    1   LCK-3
....................uUggaucuuguuugacagca       12   1   JCK-2
....................ucuggaucCuguuugacagca      1    1   JCK-2
....................ucuggaucuuguuugacGgca      2    1   JCK-1
....................ucuggaucuuguuugCcagca      1    1   JCK-1
....................ucuggaucuuguuugCcagcac     1    1   LT-2
....................ucuggaucuuguuugacagcac     27   0   LT-1
....................ucuggaucuuguuugacagcac     19   0   LCK-3
....................ucuggaucuuguuugacagcac     22   0   JCK-3
....................ucuggaucuuguuugacagcaU     2    1   LT-2
....................ucuggaucuuguuugacagcac     24   0   LT-3
....................ucuggaucuuguuugacagcac     50   0   JCK-2
....................ucuggaucuuguuugacagcac     34   0   JCK-1
....................ucuggaucuuguuugacagcaU     1    1   LT-1
....................ucuggaucuuguuugacagcac     17   0   LT-2
....................ucuggaucuuguuugacagcacu    3    0   LT-1
....................ucuggaucuuguuugacagcacu    2    0   JCK-1
....................ucuggaucuuguuugacagcacu    5    0   JCK-2
....................ucuggaucuuguuugacagcaUu    1    1   LT-3
....................ucuggaucuuguuugacagcacu    2    0   JCK-3
....................ucuggaucuuguuugacagcacuc   2    0   LT-3
....................ucuggaucuuguuugacagcacuc   1    0   JCK-2
....................ucuggaucuuguuugacagcacuc   1    0   JCK-3
....................ucuggaucuuguuugacagcacuc   1    0   JCK-1
.....................cuggaucuuguuugacagc       7    0   JCK-2
.....................cuggaucuuguuugacagc       2    0   JCK-3
.....................cuggaucuuguuugacagc       13   0   LT-2
.....................cuggaucuuguuugacagc       1    0   LT-3
.....................cuggaucuuguuugacagc       1    0   JCK-1
.....................cuggaucuuguuugacagc       5    0   LT-1
.....................cuggaucuuguuugacagc       5    0   LCK-3
.....................cuggaucuuguuugacagcU      5    1   LT-2
.....................cuggaucuuguuugCcagca      1    1   JCK-1
.....................cuggaucuuguuugacagca      11   0   LT-2
.....................cuggaucuuguuugacagca      58   0   JCK-1
.....................cuggaucuuguuugacagca      15   0   LT-3
.....................cuggaucuuguuugacagca      61   0   JCK-2
.....................cuggaucuuguuugacagca      12   0   LCK-3
.....................cuggaucuuguuugacagcU      4    1   JT-3
.....................cuggaucuuguuugacagcU      4    1   JT-2
.....................cuggaucuuguuugacagcU      1    1   JCK-2
.....................cuggaucuuguuugacagca      9    0   LT-1
.....................cuggaucuuguuugacagcU      1    1   JCK-3
.....................cuggaucuuguuugacagca      51   0   JCK-3
.....................cuggaucuuguuugacagcU      1    1   LT-3
.....................cuggaucuuguuugacagcac     4    0   LT-2
.....................cuggaucuuguuugacagcac     1    0   LCK-3
.....................cuggaucuuguuugacagcac     3    0   LT-3
.....................cuggaucuuguuugacagcacu    4    0   LT-1
.....................cuggaucuuguuugacagcaUu    1    1   JCK-2
.....................cuggaucuuguuugacagcacu    46   0   JCK-2
.....................cuggaucuuguuugacagcacu    15   0   LCK-3
.....................cuggaucuuguuugacagcaUu    2    1   JCK-3
.....................cuggaucuuguuugacagcacu    7    0   LT-2
.....................cuggaucuuguuugacagcacu    17   0   JCK-1
.....................cuggaucuuguuugacagcacu    22   0   JCK-3
.....................cuggaucuuguuugacagcacu    13   0   LT-3
```

```
                      Mature                                                   Star
cgcauguugcucguuuaccuucuggaucuuguuugacagcacucccaacguuguuguuuggaugacauagaagugcugucgaacaugauccagaaggugcugucgaacaacauacaaaaagggguuuacuuuacguaccgcccaucacgaucuucuucuucuucuucuuccugaaagaguaucaaacaugggguuucuguuuguuucucuuuuucuuuuuuaaaguagauuuacauguuaacuguauugc
.....................cuggaucuuguuugacagcacuc...........................................................................................................................................................................................................   1      0    JCK-1
.....................cuggaucuuguuugacagcacuc...........................................................................................................................................................................................................   1      0    JCK-2
.....................cuggaucuuguuugacagcacuc...........................................................................................................................................................................................................   3      0    JCK-3
......................uggaucuuguuugCcagc...............................................................................................................................................................................................................   6      1    LT-1
......................uggaucuuguuugacagc...............................................................................................................................................................................................................   1573   0    LCK-2
......................uggCucuuguuugacagc...............................................................................................................................................................................................................   7      1    LT-3
......................uNgaucuuguuugacagc...............................................................................................................................................................................................................   1      1    LT-3
......................uggaucuuguuugacaCc...............................................................................................................................................................................................................   2      1    JCK-3
......................ugAaucuuguuugacagc...............................................................................................................................................................................................................   3      1    LCK-3
......................uNgaucuuguuugacagc...............................................................................................................................................................................................................   1      1    JCK-1
......................uggCucuuguuugacagc...............................................................................................................................................................................................................   3      1    LCK-2
......................uggaucuuguuNgacagc...............................................................................................................................................................................................................   1      1    JT-1
......................uggaucuuAuuugacagc...............................................................................................................................................................................................................   3      1    LT-2
......................uggaucuuguuuAacagc...............................................................................................................................................................................................................   1      1    LCK-3
......................uggaucuCguuugacagc...............................................................................................................................................................................................................   1      1    LT-1
......................uUgaucuuguuugacagc...............................................................................................................................................................................................................   1      1    LCK-3
......................uggaucuuguuugacagU...............................................................................................................................................................................................................   9      1    LT-1
......................uNgaucuuguuugacagc...............................................................................................................................................................................................................   2      1    LCK-1
......................ugUaucuuguuugacagc...............................................................................................................................................................................................................   1      1    LCK-3
......................uggaucuugCuugacagc...............................................................................................................................................................................................................   1      1    LT-1
......................uggaucuuguuugacagA...............................................................................................................................................................................................................   4      1    LCK-3
......................ugUaucuuguuugacagc...............................................................................................................................................................................................................   1      1    LT-2
......................uggCucuuguuugacagc...............................................................................................................................................................................................................   3      1    JT-1
......................uggaucuuguuNgacagc...............................................................................................................................................................................................................   1      1    JCK-2
......................uggNucuuguuugacagc...............................................................................................................................................................................................................   1      1    JT-2
......................uggaucuuguuugacagU...............................................................................................................................................................................................................   1      1    JCK-1
......................uggaucuuguuugacagc...............................................................................................................................................................................................................   2328   0    LT-3
......................uCgaucuuguuugacagc...............................................................................................................................................................................................................   1      1    LT-1
......................uggCucuuguuugacagc...............................................................................................................................................................................................................   2      1    JT-3
......................uggaucuuguuugCcagc...............................................................................................................................................................................................................   15     1    LCK-3
......................uUgaucuuguuugacagc...............................................................................................................................................................................................................   1      1    JCK-2
......................uAgaucuuguuugacagc...............................................................................................................................................................................................................   1      1    LCK-2
......................uggaucuuguuuUacagc...............................................................................................................................................................................................................   1      1    JT-2
......................uNgaucuuguuugacagc...............................................................................................................................................................................................................   1      1    JT-1
......................uggauNuuguuugacagc...............................................................................................................................................................................................................   1      1    LCK-1
......................uggaucuuAuuugacagc...............................................................................................................................................................................................................   1      1    LCK-2
......................uAgaucuuguuugacagc...............................................................................................................................................................................................................   5      1    LT-2
......................uggaucuuguuugaNagc...............................................................................................................................................................................................................   11     1    LCK-1
......................uggaucuuguGugacagc...............................................................................................................................................................................................................   1      1    LT-2
......................ugUaucuuguuugacagc...............................................................................................................................................................................................................   1      1    JCK-1
......................uggaucuuguuugacagG...............................................................................................................................................................................................................   1      1    JCK-1
......................uggaucuuguuugacagc...............................................................................................................................................................................................................   1255   0    JCK-3
......................uggaucuuguuugaGagc...............................................................................................................................................................................................................   4      1    LCK-1
......................uggaucuuguuugacagU...............................................................................................................................................................................................................   13     1    LCK-3
......................uUgaucuuguuugacagc...............................................................................................................................................................................................................   1      1    LT-3
......................uggaucuuguuugCcagc...............................................................................................................................................................................................................   2      1    LCK-2
......................uggCucuuguuugacagc...............................................................................................................................................................................................................   2      1    JT-2
......................ugAaucuuguuugacagc...............................................................................................................................................................................................................   1      1    LT-1
......................uggaucuuguuugacagc...............................................................................................................................................................................................................   1080   0    JCK-1
......................uggaucuuguuugCcagc...............................................................................................................................................................................................................   4      1    LT-3
......................uNgaucuuguuugacagc...............................................................................................................................................................................................................   1      1    JCK-3
......................ugAaucuuguuugacagc...............................................................................................................................................................................................................   2      1    LCK-1
......................uggaucuuAuuugacagc...............................................................................................................................................................................................................   3      1    LT-1
......................uggaucCuguuugacagc...............................................................................................................................................................................................................   1      1    LT-3
......................uggaucuuguuugacCgc...............................................................................................................................................................................................................   2      1    JCK-2
......................uggaucuuguuugacagc...............................................................................................................................................................................................................   1810   0    JCK-2
......................uggaucuuguuugCcagc...............................................................................................................................................................................................................   2      1    LCK-1
......................uggaucuuguuugacagA...............................................................................................................................................................................................................   1      1    JCK-3
......................ugNaucuuguuugacagc...............................................................................................................................................................................................................   7      1    LT-2
......................uNgaucuuguuugacagc...............................................................................................................................................................................................................   1      1    LCK-3
......................uggaucuuguuugacagA...............................................................................................................................................................................................................   6      1    LT-3
......................uggaucuugCuugacagc...............................................................................................................................................................................................................   1      1    JCK-1
......................uggaucuuAuuugacagc...............................................................................................................................................................................................................   1      1    LT-3
......................ugNaucuuguuugacagc...............................................................................................................................................................................................................   1      1    LCK-1
......................uggaucuuguuugCcagc...............................................................................................................................................................................................................   4      1    JCK-2
......................uggaucuuguuugCcagc...............................................................................................................................................................................................................   24     1    LT-2
......................uggaucuuguuugacagU...............................................................................................................................................................................................................   1      1    JCK-3
......................uggaucuuguuugacagU...............................................................................................................................................................................................................   21     1    LT-2
......................uggaucuuguuugacagA...............................................................................................................................................................................................................   3      1    JCK-1
......................uUgaucuuguuugacagc...............................................................................................................................................................................................................   1      1    LT-2
```

Mature **Star**

cgcauguugcucguuuaccuucuggaucuuguuugacagcacucccaacguuguuguuuggaugacauagaagugcugucgaacaugauccagaaggugcugucgaacaacauacaaaaaggggguuuacuuuacguaccgcccaucacgaucuucuucuucuucuucuuccugaaagaguaucaaacaugggguuucuguuuguuucucuuuuucuuuuuuaaaguagauuuacauguuaacuguauugc

......................uggCucuuguuugacagc	2	1	LCK-1
......................uggaucuuguuugCcagc	1	1	JCK-3
......................uggaucuuguuugacagc	419	0	JT-3
......................uggaucuuguuugacagA	1	1	LCK-1
......................uggaucuuguuugacagc	5296	0	LT-2
......................ugAaucuuguuugacagc	1	1	JCK-2
......................uggaucuuguuugacagc	2286	0	LT-1
......................uggaucuuguuugNcagc	6	1	LCK-1
......................uggaucuuguuNgacagc	1	1	JCK-1
......................uNgaucuuguuugacagc	3	1	JCK-2
......................uAgaucuuguuugacagc	1	1	LT-1
......................uggaucuuguuugacGgc	1	1	LT-2
......................uggCucuuguuugacagc	10	1	LCK-3
......................uggaucuugCuugacagc	1	1	JCK-2
......................uggCucuuguuugacagc	2	1	JCK-2
......................Cggaucuuguuugacagc	1	1	LT-3
......................uggaucuuguuugacagU	3	1	JCK-2
......................uggaucuuguuugCcagc	3	1	JCK-1
......................uAgaucuuguuugacagc	4	1	LCK-3
......................uggaucuAguuugacagc	1	1	LT-1
......................uggCucuuguuugacagc	1	1	JCK-1
......................uggaucuuguuugacagc	1447	0	LCK-1
......................uggaucuuguCugacagc	2	1	JCK-2
......................uggCucuuguuugacagc	1	1	JCK-3
......................uggaucuuguuugGcagc	2	1	LCK-1
......................uggaucuuguuugacagc	6351	0	LCK-3
......................ugAaucuuguuugacagc	3	1	LT-2
......................uggGucuuguuugacagc	1	1	LCK-3
......................uggaucuuguuugacCgc	2	1	LCK-3
......................ugAaucuuguuugacagc	1	1	LT-3
......................uggaucuuguuuNacagc	3	1	LCK-1
......................uggaucuuguuugacCgc	1	1	JT-1
......................uggaucuuguuugacCgc	2	1	LCK-2
......................uggCucuuguuugacagc	5	1	LT-1
......................uAgaucuuguuugacagc	4	1	LT-3
......................uggaucuuguuugacCgc	2	1	LT-1
......................uggCucuuguuugacagc	53	1	LT-2
......................uggNucuuguuugacagc	1	1	JCK-1
......................uggaucuuguuugacagc	353	0	JT-2
......................uggaucuuAuuugacagc	1	1	JCK-1
......................uggGucuuguuugacagc	1	1	LT-2
......................uggaucuuguuuAacagc	1	1	LCK-2
......................uggaucuuguuugacagU	12	1	LT-3
......................uggaucuuguuugacCgc	6	1	LT-2
......................uggaucuuguuugacagc	520	0	JT-1
......................Cggaucuuguuugacagc	1	1	LT-2
......................uggaucuuguuugacagA	7	1	LT-2
......................uggaucuuguuugacagca	3565	0	LCK-1
......................uggaucuuguuugGcagca	1	1	LT-2
......................uUgaucuuguuugacagca	1	1	LT-3
......................uggaucuCguuugacagca	1	1	JT-1
......................uNgaucuuguuugacagca	1	1	LCK-3
......................uggaucuuguuugGcagca	2	1	LCK-1
......................uggaucuuguuugCcagca	3	1	LCK-1
......................uggaucuuguuugacagcG	15	1	LT-2
......................uggaucuuguuugacagUa	29	1	LT-2
......................uggaucuuguuugCcagca	2	1	LT-3
......................uggaucuuguuugacagca	3109	0	LCK-3
......................uggaucuuguuugacagAa	1	1	LT-3
......................uggaucuuguuugacagUa	12	1	JCK-1
......................uggaucuCguuugacagca	1	1	LT-1
......................uggaucuuguuugacagca	980	0	JT-2
......................uggaucuuguuugCcagca	6	1	LCK-2
......................uggaucuuUuuugacagca	1	1	LCK-2
......................uggaucuuguuugacagcU	1372	1	LCK-1
......................uggaucuuguuugacagUa	11	1	JT-2
......................uggaucuuguuugCcagca	2	1	LCK-3
......................uNgaucuuguuugacagca	2	1	JCK-2
......................uggCucuuguuugacagca	3	1	JCK-3
......................uggaucuuguuugacagcC	6	1	JCK-3

```
                      Mature                                                   Star
cgcauguugcucguuuaccuucuggaucuuguuugacagcacucccaacguuguuguuuggaugacauagaagugcugucgaacaugauccagaaggugcugucgaacaacauacaaaaggggguuuacuuuacguaccgcccaucacgaucuucuucuucuucuucuuccugaaagaguaucaaacauggggguuucuguuuguuucucuuuuucuuuuuuaaaguagauuuacauguuaacuguauugc
......................uggaucuuguuugacCgca.................................................................................................................................................................................................. 1     1    LT-2
......................uggaucuuguuugacagcG.................................................................................................................................................................................................. 11    1    LT-3
......................uggaucuuguuugacagcU.................................................................................................................................................................................................. 1503  1    LCK-2
......................uggaucuuguuugacagcC.................................................................................................................................................................................................. 8     1    LCK-2
......................uUgaucuuguuugacagca.................................................................................................................................................................................................. 5     1    JT-2
......................uggaucuuguuugNcagca.................................................................................................................................................................................................. 9     1    LCK-1
......................uggaucuugNuugacagca.................................................................................................................................................................................................. 1     1    LCK-1
......................uggaucuuguuugacagUa.................................................................................................................................................................................................. 10    1    JT-3
......................uggaucuuguuugacCgca.................................................................................................................................................................................................. 2     1    LCK-1
......................ugNaucuuguuugacagca.................................................................................................................................................................................................. 9     1    LT-2
......................uggGucuuguuugacagca.................................................................................................................................................................................................. 1     1    JCK-1
......................uggaucuuguuugacagUa.................................................................................................................................................................................................. 11    1    LT-3
......................uggaucuuguuugacagUa.................................................................................................................................................................................................. 3     1    LT-1
......................uAgaucuuguuugacagca.................................................................................................................................................................................................. 3     1    LCK-3
......................uggaucuuguuugacagcU.................................................................................................................................................................................................. 381   1    JT-3
......................uggaucuuguuugacagca.................................................................................................................................................................................................. 2333  0    LT-2
......................uggCucuuguuugacagca.................................................................................................................................................................................................. 7     1    LCK-2
......................uggCucuuguuugacagca.................................................................................................................................................................................................. 2     1    LT-1
......................uggaucuuguuugacagca.................................................................................................................................................................................................. 1490  0    LT-3
......................uggaucuuguuugacagca.................................................................................................................................................................................................. 1426  0    JT-1
......................uggaucuuguuugacCgca.................................................................................................................................................................................................. 1     1    JCK-3
......................ugUaucuuguuugacagca.................................................................................................................................................................................................. 1     1    LCK-2
......................uggaucuuguuGgacagca.................................................................................................................................................................................................. 9     1    LCK-3
......................uggaucuuguuugacagcC.................................................................................................................................................................................................. 14    1    LT-1
......................uggaucuuguuugacagcC.................................................................................................................................................................................................. 5     1    JCK-2
......................uggaucuuguuugacagcC.................................................................................................................................................................................................. 20    1    LT-2
......................uggaucuuAuuugacagca.................................................................................................................................................................................................. 1     1    LCK-2
......................uAgaucuuguuugacagca.................................................................................................................................................................................................. 1     1    LT-2
......................uggCucuuguuugacagca.................................................................................................................................................................................................. 12    1    LT-2
......................uggaucuuguuugacagcG.................................................................................................................................................................................................. 2     1    JCK-3
......................uggaucuCguuugacagca.................................................................................................................................................................................................. 1     1    LCK-2
......................uggaucuuguuugacCgca.................................................................................................................................................................................................. 2     1    JT-3
......................uggaucuuguuugacagcU.................................................................................................................................................................................................. 718   1    JCK-1
......................uggCucuuguuugacagca.................................................................................................................................................................................................. 2     1    JCK-1
......................uggaucuuguuugacagcU.................................................................................................................................................................................................. 2081  1    LT-3
......................uggaucuuguuugGcagca.................................................................................................................................................................................................. 1     1    JT-2
......................uggCucuuguuugacagca.................................................................................................................................................................................................. 5     1    JT-1
......................uggaucuuAuuugacagca.................................................................................................................................................................................................. 1     1    LCK-3
......................uggGucuuguuugacagca.................................................................................................................................................................................................. 1     1    LCK-2
......................uggCucuuguuugacagca.................................................................................................................................................................................................. 4     1    LCK-3
......................uggaucuuguuugacagca.................................................................................................................................................................................................. 858   0    LT-1
......................uggauUuuguuugacagca.................................................................................................................................................................................................. 1     1    LT-2
......................uggaucuuguuugacagcU.................................................................................................................................................................................................. 1354  1    LT-1
......................uggaucuCguuugacagca.................................................................................................................................................................................................. 1     1    LT-2
......................uggaucuuguuugacagcG.................................................................................................................................................................................................. 12    1    LT-1
......................uggaucuuguuuUacagca.................................................................................................................................................................................................. 1     1    LCK-3
......................uggaucuuguuugaGagca.................................................................................................................................................................................................. 4     1    LCK-1
......................uggaucuuguuugacagcC.................................................................................................................................................................................................. 21    1    LT-3
......................uggaCcuuguuugacagca.................................................................................................................................................................................................. 1     1    LT-2
......................uggaucuuguuugacagcC.................................................................................................................................................................................................. 2     1    JCK-1
......................uggaucuuguuugacagca.................................................................................................................................................................................................. 1294  0    JT-3
......................uggauNuuguuugacagca.................................................................................................................................................................................................. 10    1    LCK-1
......................uAgaucuuguuugacagca.................................................................................................................................................................................................. 1     1    LCK-2
......................uggaucGuguuugacagca.................................................................................................................................................................................................. 1     1    LCK-2
......................uUgaucuuguuugacagca.................................................................................................................................................................................................. 5     1    JT-3
......................uggaucuuguuugacCgca.................................................................................................................................................................................................. 1     1    JCK-1
......................uggaucCuguuugacagca.................................................................................................................................................................................................. 1     1    LT-3
......................uggaucuuguuugCcagca.................................................................................................................................................................................................. 5     1    JCK-2
......................uggaucuuguuugacagUa.................................................................................................................................................................................................. 24    1    JCK-2
......................uggCucuuguuugacagca.................................................................................................................................................................................................. 3     1    JT-2
......................uggaucuuguuugacagUa.................................................................................................................................................................................................. 11    1    JCK-3
......................uggaucuuguuugacagca.................................................................................................................................................................................................. 1862  0    JCK-2
......................uggaucuuguuugacagcG.................................................................................................................................................................................................. 2     1    JT-2
......................uggaucuuguuugacagcU.................................................................................................................................................................................................. 279   1    JT-2
......................uggCucuuguuugacagca.................................................................................................................................................................................................. 4     1    JCK-2
......................uAgaucuuguuugacagca.................................................................................................................................................................................................. 1     1    LT-3
......................uggaucuuguuugacagca.................................................................................................................................................................................................. 1154  0    JCK-1
......................uggaucuuguuugacagcC.................................................................................................................................................................................................. 21    1    LCK-3
......................uggCucuuguuugacagca.................................................................................................................................................................................................. 5     1    LCK-1
......................uggaucuuguuuNacagca.................................................................................................................................................................................................. 2     1    LCK-1
```

Mature Star

cgcauguugcucguuuaccuucuggaucuuguuugacagcacucccaacguuguuguuuggaugacauagaagugcugucgaacaugauccagaaggugcugucgaacaacauacaaaaggggguuuacuuuacguaccgcccaucacgaucuucuucuucuucuucuuccugaaagaguaucaaacauggggguuucuguuuguuucucuuuuucuuuuuuaaaguagauuuacauguuaacuguauugc

ugAaucuuguuugacagca	1	1	JCK-3
uggCucuuguuugacagca	2	1	LT-3
uggaucuuguuugCcagca	2	1	JCK-1
ugNaucuuguuugacagca	5	1	LCK-1
uggaucuuguuugacagUa	18	1	LCK-3
uggaucuuguuugacagca	3633	0	LCK-2
ugAaucuuguuugacagca	1	1	JT-1
uggaucuuguuugacagcU	2784	1	LT-2
uggaucuuguuugacagcU	4465	1	LCK-3
uggaucuuguuugacCgca	3	1	LCK-3
uggaucuuguuugacagcU	835	1	JCK-3
uggauAuuguuugacagca	1	1	JCK-3
uggaucuuguuugacCgca	2	1	LT-3
uggaucuuguuugaNagca	11	1	LCK-1
uggaucuuguuugacagcC	3	1	JT-1
uggCucuuguuugacagca	2	1	JT-3
uggaucuuguuugacCgca	1	1	JT-1
uggaucuuguuNgacagca	5	1	JCK-1
uggaucuuguuugacagcC	7	1	LCK-1
uggNucuuguuugacagca	1	1	LCK-1
ugAaucuuguuugacagca	1	1	LT-3
uggaucuuguuugaAagca	1	1	LCK-3
uggaucuugCuugacagca	1	1	JT-1
uggaucuuguuugCcagca	1	1	LT-1
uggaucuuguuugacagUa	1	1	LCK-2
uggaucuuguuugCcagca	11	1	LT-2
uggaucuuguuugacagca	1105	0	JCK-3
uNgaucuuguuugacagca	1	1	JCK-3
uggaucCuguuugacagca	1	1	LT-2
uggaucuuguuugacagcU	735	1	JT-1
uNgaucuuguuugacagca	3	1	JT-1
uggaucuuguuNgacagca	2	1	JT-1
uggaucuuguuugacagcG	2	1	JCK-2
uggaucuuguuugacCgca	2	1	JCK-2
uAgaucuuguuugacagca	1	1	JT-1
uNgaucuuguuugacagca	2	1	JCK-1
uggaucGuguuugacagca	1	1	JT-3
Cggaucuuguuugacagca	1	1	LCK-3
uggaucuuguuugacagcU	1214	1	JCK-2
uggaucuuguuugacagcG	11	1	LCK-3
uggaucuuguuugacagcG	6	1	JCK-1
uggaucuuguuugaAagca	1	1	LCK-2
uggaucCuguuugacagca	1	1	LCK-1
uggaucuuguuNgacagca	1	1	JT-2
uggaucuuguuugacagcCc	2	1	LT-1
uggaucuuguuugacagcaA	3	1	JCK-1
uggaucuuguuugCcagcac	1	1	LT-3
uggaucuuguuugacagcUc	15	1	JCK-2
uggaucuuguuugacagcac	687	0	JCK-3
uggaucuuguuugacagcaU	26	1	LT-3
uggaucuuguuugacagcaU	26	1	JCK-3
uggaucuuguuugacCgcac	1	1	LT-2
uggaucuuguuugacagcUc	7	1	JCK-3
uggaucuuguuugacagcaU	66	1	LCK-3
uggaucuuguuugacGgcac	1	1	LT-2
uggaucuuguuugacagcaG	1	1	LT-1
uggCucuuguuugacagcac	1	1	JCK-1
uggaucuuguuugacagcaA	1	1	JCK-2
uggaucuuguuugacagcac	249	0	JT-3
uggGucuuguuugacagcac	2	1	LT-2
uggaucuugCuugacagcac	1	1	LT-2
uggaucuuguuugacagcac	170	0	JT-2
uggaucuuguuugacagcaU	22	1	LT-1
uggaCcuuguuugacagcac	1	1	JCK-3
uAgaucuuguuugacagcac	1	1	LCK-1
uggaCcuuguuugacagcac	1	1	JCK-2
uggCucuuguuugacagcac	1	1	LCK-1
uggaucuuguuugGcagcac	1	1	LT-3
uggaucuuguuugCcagcac	1	1	LCK-3
uNgaucuuguuugacagcac	1	1	LCK-3

```
                      Mature                           Star
cgcauguugcucguuuaccuucuggaucuuguuugacagcacucccaacguuguuguuuggaugacauagaagugcugucgaacaugauccagaaggugcugucgaacaacauacaaaaaggggguuuacuuuacguaccgcccaucacgaucuucuucuucuucuucuuccugaaagaguaucaaacaugggguuucuguuuguuucucuuuucuuuuuuaaaguagauuuacauguuaacuguauugc
......................uggaucuuguuugCcagcac.......   4     1   LT-2
......................uAgaucuuguuugacagcac.......   1     1   LCK-3
......................uggaucuuguuugacagcac.......   216   0   JT-1
......................uggCucuuguuugacagcac.......   2     1   JT-3
......................Aggaucuuguuugacagcac.......   1     1   LCK-3
......................uggaucuuUuuugacagcac.......   1     1   LT-3
......................uggaucuuguuugGcagcac.......   1     1   LCK-1
......................uAgaucuuguuugacagcac.......   1     1   LT-1
......................uggaucuuguuugacagcGc.......   1     1   LCK-3
......................uggaucuuguuugacagcUc.......   6     1   JCK-1
......................uggaucuuguuNgacagcac.......   1     1   JT-2
......................uggCucuuguuugacagcac.......   5     1   LCK-3
......................uggaucuuguCugacagcac.......   1     1   JCK-2
......................uggaucuuguuugacagcaU.......   16    1   JT-1
......................uggaucuuguuugacagcac.......   545   0   LCK-1
......................uggaucuuguuugacagcUc.......   77    1   LT-2
......................uggaucuuguuugCcagcac.......   2     1   JT-1
......................uggaucuuguuugacagcCc.......   1     1   JT-1
......................uggaucuuguuugacagcac.......   1318  0   LCK-3
......................uggaucuuguuugacagcac.......   666   0   LT-1
......................uggaucuuguuugacCgcac.......   2     1   JCK-2
......................uggaucuuguuugacagcUc.......   36    1   LT-1
......................uggaucuuguuugCcagcac.......   1     1   JCK-3
......................uggCucuuguuugacagcac.......   1     1   LT-1
......................uggaucuuguuugacagcCc.......   1     1   LT-2
......................uggaucuuguuugacagUac.......   1     1   JCK-3
......................uggaucuuguuugCcagcac.......   1     1   JCK-1
......................uggaucuuguuugacagcaA.......   1     1   LCK-2
......................uggaucuuguuugacagcaU.......   8     1   JCK-1
......................uggCucuuguuugacagcac.......   2     1   JCK-2
......................uggaucuuguuugacagcaA.......   4     1   JCK-3
......................uggauNuuguuugacagcac.......   1     1   LCK-1
......................ugNaucuuguuugacagcac.......   3     1   LT-2
......................uggaucuuguuuAacagcac.......   1     1   LCK-2
......................uggaucuuguuugacagcac.......   525   0   LCK-2
......................ugAaucuuguuugacagcac.......   1     1   JCK-1
......................uggaucuuguuugacagcUc.......   51    1   LT-3
......................uggaucuuguuugacagcac.......   982   0   LT-2
......................uggaCcuuguuugacagcac.......   1     1   JT-1
......................uggaucuuguuugacagcUc.......   63    1   LCK-3
......................uggCucuuguuugacagcac.......   2     1   LT-3
......................uggCucuuguuugacagcac.......   9     1   LT-2
......................uggaucuuguuugacagcaA.......   1     1   JT-3
......................uggaucuuguuugacagcaU.......   26    1   LT-2
......................uggaucuuguuugCcagcac.......   3     1   JCK-2
......................uggaucuuguuugCcagcac.......   1     1   LT-1
......................uggaucuuguuugacagcUc.......   26    1   LCK-1
......................uggaucuuguuugacagcac.......   715   0   LT-3
......................uAgaucuuguuugacagcac.......   4     1   LT-2
......................uNgaucuuguuugacagcac.......   1     1   JCK-1
......................uggaucuuguuugacagcaU.......   46    1   JCK-2
......................uggaucuuguuugacagcaA.......   3     1   LT-3
......................uggaucuuguuugacagcUc.......   31    1   LCK-2
......................uggaucuuguuugNcagcac.......   2     1   LCK-1
......................uggaucuuguuugacCgcac.......   3     1   LT-3
......................uggCucuuguuugacagcac.......   1     1   LCK-2
......................uggaucuuguuugacagcac.......   586   0   JCK-1
......................uggaucuuguuugacagcNc.......   1     1   JCK-1
......................uggaucuuguuugacagcac.......   899   0   JCK-2
......................uggaucuuguuugCcagcac.......   1     1   JT-2
......................uggaucuuguuugaNagcac.......   2     1   LCK-1
......................uggaucuuCuuugacagcac.......   1     1   LCK-3
......................uggaucuuguuugacagcaN.......   2     1   JCK-1
......................ugAaucuuguuugacagcacu......   4     1   LCK-3
......................uggaucuuguuugacagcaAu......   3     1   JCK-3
......................uggaucuuAuuugacagcacu......   1     1   LCK-1
......................uggaucuuguuugacagUacu......   18    1   JCK-1
......................uggaucuuguuuAacagcacu......   1     1   LCK-1
......................uggaucuuguuugacagcacG......   12    1   LT-3
......................uggaucCuguuugacagcacu......   1     1   LCK-1
```

```
                      Mature                                                  Star
cgcauguugcucguuuaccuucuggaucuuguuugacagcacucccaacguuguuguuuggaugacauagaagugcugucgaacaugauccagaaggugcugucgaacaacauacaaaaagggguuuacuuuacguaccgcccaucacgaucuucuucuucuucuucuuccugaaagaguaucaaacaugggguuucuguuuguuucucuuuuucuuuuuuaaaguagauuuacauguuaacuguauugc
......................ugCaucuuguuugacagcacu............................................................................................................................................................................................................... 1 1 LCK-3
......................uggaucuuAuuugacagcacu............................................................................................................................................................................................................... 4 1 LCK-3
......................uggaucuuCuuugacagcacu............................................................................................................................................................................................................... 1 1 JCK-2
......................uggCucuuguuugacagcacu............................................................................................................................................................................................................... 200 1 LT-2
......................uggaucuuguuugacagUacu............................................................................................................................................................................................................... 1 1 JT-2
......................uggaucuuguuugacagcacu............................................................................................................................................................................................................... 658 0 JT-2
......................uggaucuuguuugacagcacG............................................................................................................................................................................................................... 3 1 LT-2
......................uggaucuuguuuUacagcacu............................................................................................................................................................................................................... 2 1 JCK-3
......................uggaucuuguuugaAagcacu............................................................................................................................................................................................................... 3 1 LT-1
......................uggaucuuguuugacagcCcu............................................................................................................................................................................................................... 31 1 LT-3
......................uNgaucuuguuugacagcacu............................................................................................................................................................................................................... 3 1 LT-1
......................uggaucuuguuuUacagcacu............................................................................................................................................................................................................... 4 1 LT-1
......................uggaucuuAuuugacagcacu............................................................................................................................................................................................................... 6 1 JCK-1
......................uggaucGuguuugacagcacu............................................................................................................................................................................................................... 2 1 JCK-1
......................uggaCcuuguuugacagcacu............................................................................................................................................................................................................... 4 1 JCK-1
......................uggaucuuguuugaNagcacu............................................................................................................................................................................................................... 17 1 LCK-1
......................ugUaucuuguuugacagcacu............................................................................................................................................................................................................... 3 1 LT-3
......................uggaucuuguuugGcagcacu............................................................................................................................................................................................................... 3 1 JCK-2
......................ugCaucuuguuugacagcacu............................................................................................................................................................................................................... 1 1 JCK-3
......................uggaucuugCuugacagcacu............................................................................................................................................................................................................... 1 1 LT-3
......................uggaucuuAuuugacagcacu............................................................................................................................................................................................................... 20 1 JCK-2
......................uggaucuuguuugacGgcacu............................................................................................................................................................................................................... 1 1 JT-3
......................uggaucuuguuugacCgcacu............................................................................................................................................................................................................... 28 1 JCK-3
......................uggaucuuguuugCcagcacu............................................................................................................................................................................................................... 36 1 LCK-3
......................uggauUuuguuugacagcacu............................................................................................................................................................................................................... 4 1 JCK-3
......................uggaucuuguuugCcagcacu............................................................................................................................................................................................................... 159 1 JCK-2
......................uggaucuuguuugacCgcacu............................................................................................................................................................................................................... 5 1 LCK-2
......................uNgaucuuguuugacagcacu............................................................................................................................................................................................................... 1 1 LCK-2
......................uggaucuuguuuAacagcacu............................................................................................................................................................................................................... 3 1 LT-1
......................uggaucuuguCugacagcacu............................................................................................................................................................................................................... 5 1 JT-2
......................uggaucuuguuugacagcaUu............................................................................................................................................................................................................... 1426 1 JCK-1
......................uggCucuuguuugacagcacu............................................................................................................................................................................................................... 7 1 JT-1
......................ugUaucuuguuugacagcacu............................................................................................................................................................................................................... 1 1 LCK-2
......................uggaucuuguuugGcagcacu............................................................................................................................................................................................................... 1 1 LT-3
......................uggaucuuguuugacagcGcu............................................................................................................................................................................................................... 3 1 LT-2
......................uggaucuuguuugacagcacu............................................................................................................................................................................................................... 4848 0 LCK-1
......................uggaucuuguuugacCgcacu............................................................................................................................................................................................................... 12 1 LT-1
......................uggaucuuguuugacagcacu............................................................................................................................................................................................................... 23834 0 LT-1
......................uggauUuuguuugacagcacu............................................................................................................................................................................................................... 11 1 JCK-2
......................uggaucCuguuugacagcacu............................................................................................................................................................................................................... 2 1 LT-3
......................uggaucuCguuugacagcacu............................................................................................................................................................................................................... 3 1 LT-3
......................uggaucuuguuugacagcCcu............................................................................................................................................................................................................... 4 1 LCK-1
......................uggCucuuguuugacagcacu............................................................................................................................................................................................................... 118 1 JCK-3
......................ugAaucuuguuugacagcacu............................................................................................................................................................................................................... 2 1 LCK-1
......................uggaucuuguuugacagcacu............................................................................................................................................................................................................... 5029 0 LCK-2
......................uggaucuuguuugacagcaAu............................................................................................................................................................................................................... 3 1 JCK-1
......................uggaucuugCuugacagcacu............................................................................................................................................................................................................... 2 1 JCK-1
......................Cggaucuuguuugacagcacu............................................................................................................................................................................................................... 1 1 JT-3
......................uNgaucuuguuugacagcacu............................................................................................................................................................................................................... 4 1 JT-1
......................uggaucuuguCugacagcacu............................................................................................................................................................................................................... 1 1 LT-3
......................uggaucuuguuugacagcNcu............................................................................................................................................................................................................... 22 1 JCK-1
......................uggaucuuguuugacagcCcu............................................................................................................................................................................................................... 74 1 JCK-2
......................uggaucuuguCugacagcacu............................................................................................................................................................................................................... 1 1 LCK-2
......................uNgaucuuguuugacagcacu............................................................................................................................................................................................................... 66 1 JCK-1
......................uggaucuuguuuNacagcacu............................................................................................................................................................................................................... 1 1 LCK-1
......................Cggaucuuguuugacagcacu............................................................................................................................................................................................................... 11 1 JCK-2
......................uggaucuuguCugacagcacu............................................................................................................................................................................................................... 6 1 JCK-1
......................uggCucuuguuugacagcacu............................................................................................................................................................................................................... 39 1 LCK-3
......................uggaucuuguuugacagcaNu............................................................................................................................................................................................................... 42 1 JCK-1
......................uCgaucuuguuugacagcacu............................................................................................................................................................................................................... 1 1 JCK-3
......................ugUaucuuguuugacagcacu............................................................................................................................................................................................................... 2 1 LT-2
......................uggaucuuguuugacCgcacu............................................................................................................................................................................................................... 7 1 LCK-3
......................uggaucuuguuugacagUacu............................................................................................................................................................................................................... 5 1 LT-3
......................uggaucuuguuugacagcaAu............................................................................................................................................................................................................... 1 1 LT-1
......................uggaucuuguuugacagcaUu............................................................................................................................................................................................................... 68 1 LT-2
......................uggaCcuuguuugacagcacu............................................................................................................................................................................................................... 3 1 LT-1
......................uggaucuuguuuAacagcacu............................................................................................................................................................................................................... 1 1 LT-3
......................uggaucuuguuugacaCcacu............................................................................................................................................................................................................... 3 1 JCK-2
......................uggaucuuguuuAacagcacu............................................................................................................................................................................................................... 1 1 LCK-3
......................uggaucuuguCugacagcacu............................................................................................................................................................................................................... 7 1 JCK-3
```

Mature **Star**

cgcauguugcucguuuaccuucuggaucuuguuugacagcacucccaacguuguuguuuggaugacauagaagugcugucgaacaugauccagaaggugcugucgaacaacauacaaaaagggguuuacuuuacguaccgcccaucacgaucuucuucuucuucuucuuccugaaagaguaucaaacaugggguuucuguuuguuucucuuuuucuuuuuuaaaguagauuuacauguuaacuguauugc

......................uggaucuuguuNgacagcacu…	1	1	JCK-2
......................uggaucuuguuugCcagcacu…	61	1	LT-1
......................uggaucuuguuugacCgcacu…	11	1	LT-3
......................uggaucuuguuugacagcacG…	16	1	JCK-2
......................uNgaucuuguuugacagcacu…	55	1	JCK-3
......................uggaucuuguuugacaCcacu…	5	1	JCK-3
......................uggNucuuguuugacagcacu…	3	1	JCK-3
......................uggaucuuguuAgacagcacu…	1	1	LCK-3
......................uCgaucuuguuugacagcacu…	1	1	LCK-3
......................uggaucuuguuugaGagcacu…	1	1	JT-1
......................uggaucuuguuuAacagcacu…	2	1	LT-2
......................uAgaucuuguuugacagcacu…	8	1	JCK-2
......................uggauUuuguuugacagcacu…	3	1	JCK-1
......................uggGucuuguuugacagcacu…	1	1	JT-1
......................uggaCcuuguuugacagcacu…	13	1	JCK-3
......................uggaucuuguuugacagcaUu…	20	1	LT-1
......................uggaCcuuguuugacagcacu…	14	1	JCK-2
......................ugAaucuuguuugacagcacu…	19	1	JCK-2
......................uggaCcuuguuugacagcacu…	1	1	LCK-3
......................uggaucuuguuugCcagcacu…	5	1	LCK-1
......................uggaucuugAuugacagcacu…	1	1	JCK-2
......................uggaucuuguuugacCgcacu…	3	1	LCK-1
......................uUgaucuuguuugacagcacu…	7	1	LT-2
......................uggaucuuguuugacCgcacu…	29	1	JCK-2
......................uggaucuuguuugacagcacG…	7	1	LCK-3
......................uggaucuuguuugacagcCcu…	2	1	JT-1
......................uggaucuuguuugacagNacu…	3	1	JCK-3
......................uggaucuuAuuugacagcacu…	4	1	JCK-3
......................uggaucuuguuGgacagcacu…	1	1	LT-3
......................uAgaucuuguuugacagcacu…	4	1	LCK-2
......................uAgaucuuguuugacagcacu…	13	1	LCK-3
......................Cggaucuuguuugacagcacu…	7	1	JCK-3
......................uggCucuuguuugacagcacu…	2	1	JT-2
......................uggaucuuguuugacagcGcu…	2	1	JCK-1
......................uNgaucuuguuugacagcacu…	1	1	LCK-1
......................uggaucuuguCugacagcacu…	3	1	LT-1
......................uggaucuuguuugacagcCcu…	16	1	LCK-3
......................uggaucuuguuugGcagcacu…	3	1	JCK-1
......................uggaucuuguuuAacagcacu…	1	1	JT-1
......................uggCucuuguuugacagcacu…	1	1	JT-3
......................uAgaucuuguuugacagcacu…	5	1	JCK-1
......................uggaucuuguuuUacagcacu…	6	1	JCK-2
......................uggaucuuUuuugacagcacu…	4	1	JCK-2
......................uAgaucuuguuugacagcacu…	15	1	JCK-3
......................ugNaucuuguuugacagcacu…	1	1	LCK-1
......................uggauGuuguuugacagcacu…	1	1	JCK-3
......................uggGucuuguuugacagcacu…	8	1	JCK-1
......................uggaucuuUuuugacagcacu…	1	1	LCK-3
......................uggaucuuguuugacagcUcu…	1	1	JCK-2
......................uggaucuuguuNgacagcacu…	1	1	JT-1
......................uggaucuuguuugacagcaUu…	21	1	JT-3
......................uUgaucuuguuugacagcacu…	2	1	LT-1
......................uggaucuuguuugCcagcacu…	105	1	JCK-3
......................ugAaucuuguuugacagcacu…	10	1	LT-2
......................uggaucuuguuuCacagcacu…	2	1	JCK-1
......................uggaucuuguuugacagcacG…	2	1	LCK-1
......................uUgaucuuguuugacagcacu…	1	1	JT-3
......................uggaucuuguuugacagAacu…	3	1	LT-1
......................uggaucuuguuugacGgcacu…	9	1	JCK-3
......................uggCucuuguuugacagcacu…	92	1	JCK-1
......................uggauNuuguuugacagcacu…	1	1	JCK-2
......................uggaucuuguuugacagAacu…	1	1	JCK-2
......................Gggaucuuguuugacagcacu…	1	1	JCK-2
......................ugUaucuuguuugacagcacu…	2	1	JCK-1
......................uggaucuuguuGgacagcacu…	10	1	JCK-1
......................uggaucCuguuugacagcacu…	10	1	JCK-1
......................uggaucuuguuugacagcacu…	20839	0	LT-2
......................uggaucCuguuugacagcacu…	2	1	LT-2
......................uggaucuuguuugUcagcacu…	2	1	JCK-2
......................uggauUuuguuugacagcacu…	1	1	LT-2

Mature **Star**

cgcauguugcucguuuaccuucuggaucuuguuugacagcacucccaacguuguuguuuggaugacauagaagugcugucgaacaugauccagaaggugcugucgaacaacauacaaaaagggguuuacuuuacguaccgcccaucacgaucuucuucuucuucuucuuccugaaagaguaucaaacaugggguuucuguuuguuucucuuuuucuuuuuuaaaguagauuuacauguuaacuguauugc

uggaucuugCuugacagcacu	6	1	JCK-3
uggaucuuguuugacagcaAu	1	1	LCK-2
uggaucuuguuugacagcCcu	17	1	JCK-3
uggaucuuguuugacagGacu	24	1	JCK-1
ugAaucuuguuugacagcacu	3	1	JCK-1
uggaucuuCuuugacagcacu	1	1	JCK-3
Cggaucuuguuugacagcacu	6	1	JCK-1
uggaucuuguuugacagcacG	13	1	JCK-3
uggaucuuguuugacagcacu	27651	0	LT-3
uggaucuuguuugacagGacu	2	1	JCK-3
uggaucuuguuugNcagcacu	15	1	LCK-1
uggaucuCguuugacagcacu	1	1	LT-1
uggaucuuguuugacagcacu	38814	0	JCK-1
ugAaucuuguuugacagcacu	8	1	JCK-3
uggaucuuguuugacCgcacu	3	1	JT-1
uggaucuuguuugCcagcacu	86	1	JCK-1
uggaucuuguuugGcagcacu	3	1	JCK-3
uggaucuuguuugacagcaUu	64	1	LT-3
uggaucuuguuugacagcacG	10	1	JCK-1
uggaucuuguuugacagcaAu	5	1	JT-2
uNgaucuuguuugacagcacu	104	1	JCK-2
uUgaucuuguuugacagcacu	2	1	LCK-3
uggaucuuguuugacagGacu	2	1	JCK-2
uggaucuuguuAgacagcacu	1	1	JCK-1
uggaucuuguuGgacagcacu	1	1	JCK-3
uggaucuugCuugacagcacu	4	1	LCK-3
uggaucuuguuAgacagcacu	1	1	JCK-2
uggaucuCguuugacagcacu	4	1	JCK-3
uggaucuuguuugacGgcacu	7	1	LT-2
uAgaucuuguuugacagcacu	22	1	LT-3
uggaucuuguuugacagcGcu	2	1	JCK-2
uggaucuuAuuugacagcacu	9	1	LT-2
Cggaucuuguuugacagcacu	3	1	LT-1
uggaucuuguCugacagcacu	6	1	JCK-2
uggaucuuguuuCacagcacu	1	1	JCK-3
uggaucuuguuugacagcaAu	10	1	JCK-2
uggaucuuguuugacagcacu	20263	0	LCK-3
uggaucuuguuNgacagcacu	121	1	JCK-1
uggCucuuguuugacagcacu	95	1	LT-3
uggaucuuguuugacagcacu	979	0	JT-3
uggaucuuguuugacagAacu	1	1	JCK-1
uggaucCuguuugacagcacu	4	1	LT-1
uggaucuuAuuugacagcacu	3	1	LT-3
uggaCcuuguuugacagcacu	4	1	LT-3
uggaucuuguuugacaCcacu	1	1	JT-1
uggaucuuguuugacagcacG	10	1	LT-1
uggaucuuguuugacaCcacu	1	1	LT-3
uggaucuuguuGgacagcacu	1	1	JCK-2
ugUaucuuguuugacagcacu	1	1	JT-1
uggCucuuguuugacagcacu	65	1	LT-1
uggaGcuuguuugacagcacu	2	1	JCK-3
uNgaucuuguuugacagcacu	2	1	LT-3
uggaucuuguuugacagcUcu	6	1	LT-2
uggCucuuguuugacagcacu	8	1	LCK-1
uggaucuuUuuugacagcacu	3	1	LT-2
uggaucuugGuugacagcacu	1	1	JCK-1
ugNaucuuguuugacagcacu	65	1	LT-2
uggaucuuguuugacagUacu	13	1	JCK-3
uggaCcuuguuugacagcacu	1	1	LCK-2
uggaucuuguGugacagcacu	2	1	LT-2
uggaucuuguuugCcagcacu	1	1	JT-3
uggaucuuguuugGcagcacu	1	1	LT-2
uggaucuuguuugGcagcacu	2	1	LT-1
uggaucuuUuuugacagcacu	1	1	LT-3
uggaucuuguuugCcagcacu	80	1	LT-2
Cggaucuuguuugacagcacu	1	1	JT-1
uggaucuugGuugacagcacu	1	1	JCK-3
uggGucuuguuugacagcacu	3	1	LT-3
uggaucuuUuuugacagcacu	6	1	JCK-3
uggaucuuguuugacagcNcu	1	1	LCK-1

```
                      Mature                                                   Star
cgcauguugcucguuuaccuucuggaucuuguuugacagcacucccaacguuguuguuuggaugacauagaagugcugucgaacaugauccagaaggugcugucgaacaacauacaaaagggguuuacuuuacguaccgcccaucacgaucuucuucuucuucuucuuccugaaagaguaucaaacaugggguuucuguuuguuucucuuuucuuuuuuaaaguagauuuacauguuaacuguauugc
......................uggaucuuguuugacagUacu............................................................................................................................................................................................................. 11     1     LT-2
......................uggaucuuguuugCcagcacu............................................................................................................................................................................................................. 5      1     JT-1
......................uggaucuugCuugacagcacu............................................................................................................................................................................................................. 4      1     LT-2
......................uggaucuuguuugacGgcacu............................................................................................................................................................................................................. 2      1     LT-1
......................uggaucuuguuugacagcaAu............................................................................................................................................................................................................. 1      1     LCK-3
......................uggNucuuguuugacagcacu............................................................................................................................................................................................................. 1      1     LCK-1
......................uggaucuCguuugacagcacu............................................................................................................................................................................................................. 10     1     JCK-2
......................uggGucuuguuugacagcacu............................................................................................................................................................................................................. 8      1     JCK-2
......................uggCucuuguuugacagcacu............................................................................................................................................................................................................. 152    1     JCK-2
......................uAgaucuuguuugacagcacu............................................................................................................................................................................................................. 14     1     LT-1
......................uggaucGuguuugacagcacu............................................................................................................................................................................................................. 1      1     LT-1
......................ugCaucuuguuugacagcacu............................................................................................................................................................................................................. 2      1     LT-2
......................uggaucuugCuugacagcacu............................................................................................................................................................................................................. 2      1     LT-1
......................Cggaucuuguuugacagcacu............................................................................................................................................................................................................. 4      1     LT-2
......................uggaucuuguuugacagcaAu............................................................................................................................................................................................................. 4      1     JT-3
......................uAgaucuuguuugacagcacu............................................................................................................................................................................................................. 30     1     LT-2
......................uggaucuuguuugGcagcacu............................................................................................................................................................................................................. 5      1     LCK-1
......................uggaCcuuguuugacagcacu............................................................................................................................................................................................................. 2      1     LT-2
......................uggaucuuguuugacGgcacu............................................................................................................................................................................................................. 3      1     LT-3
......................uggaucuCguuugacagcacu............................................................................................................................................................................................................. 3      1     LCK-3
......................uggaucuuguuuUacagcacu............................................................................................................................................................................................................. 6      1     JCK-1
......................uggaucuuguuugacagcUcu............................................................................................................................................................................................................. 1      1     LT-3
......................uggaucuuguGugacagcacu............................................................................................................................................................................................................. 15     1     JCK-1
......................uggaucuuguuugaAagcacu............................................................................................................................................................................................................. 5      1     JCK-3
......................uggauAuuguuugacagcacu............................................................................................................................................................................................................. 1      1     JCK-3
......................ugAaucuuguuugacagcacu............................................................................................................................................................................................................. 3      1     LCK-2
......................uUgaucuuguuugacagcacu............................................................................................................................................................................................................. 10     1     JCK-3
......................uggaucuuguuugacGgcacu............................................................................................................................................................................................................. 2      1     LCK-3
......................ugUaucuuguuugacagcacu............................................................................................................................................................................................................. 1      1     JCK-2
......................uggaucuuguuuAacagcacu............................................................................................................................................................................................................. 7      1     JCK-3
......................uggaucuuguuugacagcaAu............................................................................................................................................................................................................. 4      1     LT-2
......................uggaucuuguuugacagcaUu............................................................................................................................................................................................................. 55     1     JT-1
......................uggaucuuguuugacagcacG............................................................................................................................................................................................................. 1      1     JT-2
......................uggaucuuguuugacagUacu............................................................................................................................................................................................................. 34     1     JCK-2
......................uggaucuuguuugacagcCcu............................................................................................................................................................................................................. 4      1     LCK-2
......................uggaucuuguuugaGagcacu............................................................................................................................................................................................................. 8      1     LCK-1
......................uggNucuuguuugacagcacu............................................................................................................................................................................................................. 1      1     JCK-2
......................uggaucuuguuugacagcacu............................................................................................................................................................................................................. 1709   0     JT-1
......................uggGucuuguuugacagcacu............................................................................................................................................................................................................. 5      1     JCK-3
......................uggaucuuguuugacagcCcu............................................................................................................................................................................................................. 53     1     JCK-1
......................uggaucuGguuugacagcacu............................................................................................................................................................................................................. 1      1     JCK-2
......................uggaucuuguuugacagcaUu............................................................................................................................................................................................................. 2361   1     JCK-2
......................uggaucuuguuugacagcaUu............................................................................................................................................................................................................. 22     1     JT-2
......................Cggaucuuguuugacagcacu............................................................................................................................................................................................................. 9      1     LT-3
......................uggaucuuguuugacGgcacu............................................................................................................................................................................................................. 10     1     JCK-2
......................uggaucuuguuugacagcCcu............................................................................................................................................................................................................. 29     1     LT-1
......................uggaucuuAuuugacagcacu............................................................................................................................................................................................................. 6      1     LT-1
......................uggaucuuguuugacagcacu............................................................................................................................................................................................................. 48441  0     JCK-3
......................ugUaucuuguuugacagcacu............................................................................................................................................................................................................. 4      1     JCK-3
......................uggaucCuguuugacagcacu............................................................................................................................................................................................................. 8      1     JCK-3
......................uggauNuuguuugacagcacu............................................................................................................................................................................................................. 3      1     LCK-1
......................uggaucuuguuugacagcGcu............................................................................................................................................................................................................. 1      1     JT-3
......................uggaucuuguuugCcagcacu............................................................................................................................................................................................................. 7      1     LCK-2
......................uggaucuuAuuugacagcacu............................................................................................................................................................................................................. 1      1     LCK-2
......................uggaucuuguuugacagcaUu............................................................................................................................................................................................................. 37     1     LCK-2
......................uggaucuuguuuUacagcacu............................................................................................................................................................................................................. 2      1     LT-2
......................uggaucCuguuugacagcacu............................................................................................................................................................................................................. 12     1     JCK-2
......................uggaucuuguuugacCgcacu............................................................................................................................................................................................................. 19     1     JCK-1
......................uggaucuCguuugacagcacu............................................................................................................................................................................................................. 1      1     LCK-1
......................uggaucuuguuugacCgcacu............................................................................................................................................................................................................. 26     1     LT-2
......................uggaucuuCuuugacagcacu............................................................................................................................................................................................................. 1      1     JCK-1
......................uggaucuuguuugacGgcacu............................................................................................................................................................................................................. 7      1     JCK-1
......................uggaucuuguuugacagcaUu............................................................................................................................................................................................................. 36     1     LCK-1
......................uggaucuuguuugaAagcacu............................................................................................................................................................................................................. 4      1     JCK-2
......................uggaucuuguuuAacagcacu............................................................................................................................................................................................................. 6      1     JCK-1
......................uggauUuuguuugacagcacu............................................................................................................................................................................................................. 1      1     LCK-3
......................uggCucuuguuugacagcacu............................................................................................................................................................................................................. 9      1     LCK-2
......................uggaucuCguuugacagcacu............................................................................................................................................................................................................. 5      1     JCK-1
......................uggaucuugGuugacagcacu............................................................................................................................................................................................................. 1      1     JCK-2
......................uggaucuuguuugacagcCcu............................................................................................................................................................................................................. 34     1     LT-2
```

Mature   Star

```
cgcauguugcucguuuaccuucuggaucuuguuugacagcacucccaacguuguuguuuggaugacauagaagugcugucgaacaugauccagaaggugcugucgaacaacauacaaaaagggguuuacuuuacguaccgcccaucacgaucuucuucuucuucuucuuccugaaagaguaucaaacaugggguuucuguuuguuucucuuuuucuuuuuuaaaguagauuuacauguuaacuguauugc
......................uggaucuuguuugacagcacN     22     1  JCK-1
......................uggaucuuguuugacagcUcu     5      1  LT-1
......................uggaucuuguuugacaCcacu     5      1  JCK-1
......................uggaucuuNuuugacagcacu     1      1  JCK-3
......................uggaucuuUuuugacagcacu     2      1  LT-1
......................uggauAuuguuugacagcacu     2      1  JCK-2
......................uggaucuuguCugacagcacu     1      1  LT-2
......................uggaucuugCuugacagcacu     6      1  JCK-2
......................uggaucuuguuugacagcaUu     1732   1  JCK-3
......................uggaucuuguuugaAagcacu     2      1  JCK-1
......................uggauUuuguuugacagcacu     2      1  LT-3
......................uggaucuuguuuAacagcacu     9      1  JCK-2
......................uggNucuuguuugacagcacu     22     1  JCK-1
......................uggaucuuguuuCacagcacu     1      1  JCK-2
......................uggaucuuguuugacagcaUu     102    1  LCK-3
......................uggaucuuguuugacagcacu     66730  0  JCK-2
......................ugAaucuuguuugacagcacu     6      1  LT-1
......................uUgaucuuguuugacagcacu     4      1  JCK-2
......................ugAaucuuguuugacagcacu     11     1  LT-3
......................uggaucuuguuugacagcaAu     3      1  LT-3
......................uggaucuuguuugCcagcacu     67     1  LT-3
......................uggaucuuguuugacagcaGu     2      1  JCK-1
......................uggaucuuguuuUacagcacu     3      1  LT-3
......................uggaucuuguuugCcagcacu     2      1  JT-2
......................uggaucuuUuuugacagcacu     3      1  JCK-1
......................uggaucuuguuugacGgcacuc    2      1  JCK-3
......................uggaucuuguuugacCgcacuc    13     1  JCK-2
......................uggaucuuguuugacagcacuU    27     1  LT-2
......................uggaucuuguuugacagcacuG    3      1  LT-3
......................uggaucuuguuugacagcacuU    1      1  JT-2
......................uAgaucuuguuugacagcacuc    1      1  LCK-3
......................uggaCcuuguuugacagcacuc    6      1  JCK-1
......................uggaucuuguuugacagcacuc    152    0  JT-3
......................uggaucuuUuuugacagcacuc    2      1  JCK-2
......................uggaucuuguuugacagcacuG    1      1  LT-2
......................uggaucuuguuuAacagcacuc    1      1  JCK-3
......................uggCucuuguuugacagcacuc    7      1  LT-3
......................Cggaucuuguuugacagcacuc    1      1  LT-1
......................uAgaucuuguuugacagcacuc    1      1  LT-2
......................uggaucuuguuugCcagcacuc    1      1  JT-2
......................uggaucuuguuugCcagcacuc    24     1  JCK-1
......................Cggaucuuguuugacagcacuc    6      1  JCK-2
......................uggaucCuguuugacagcacuc    2      1  JCK-2
......................uggCucuuguuugacagcacuc    2      1  LCK-1
......................uggCucuuguuugacagcacuc    33     1  JCK-3
......................uAgaucuuguuugacagcacuc    1      1  LT-3
......................uggaucuuguuuUacagcacuc    1      1  JCK-3
......................uggaucuuguuugacagcacuG    11     1  JCK-1
......................uggaucuuguuugaAagcacuc    4      1  JCK-2
......................uggCucuuguuugacagcacuc    4      1  LT-1
......................uggaucuuguuuAacagcacuc    3      1  JCK-2
......................uggCucuuguuugacagcacuc    36     1  JCK-2
......................uggaucuuguuugacagcacGc    1      1  JCK-1
......................Cggaucuuguuugacagcacuc    1      1  LCK-3
......................uAgaucuuguuugacagcacuc    2      1  LT-1
......................uggauUuuguuugacagcacuc    1      1  JCK-3
......................uggaucuuguuugacagcGcuc    1      1  JCK-1
......................uggaucuuguuugacagUacuc    5      1  JCK-2
......................uUgaucuuguuugacagcacuc    1      1  JCK-3
......................ugAaucuuguuugacagcacuc    1      1  LT-1
......................uggaucuuguuugacCgcacuc    1      1  LT-1
......................ugAaucuuguuugacagcacuc    2      1  JCK-3
......................uggaucuuguuugacagcCcuc    7      1  JCK-2
......................uggaucuuguuugacagcacuN    9      1  JCK-1
......................uggaucuuguuugacagcCcuc    9      1  JCK-3
......................uggaucuuguuugCcagcacuc    2      1  LT-3
......................uggaucuCguuugacagcacuc    2      1  JCK-3
......................ugUaucuuguuugacagcacuc    1      1  JCK-2
......................uggaucuuguuugacagcaUuc    3      1  LT-2
......................uggaucuuguuugacagcacuc    520    0  LCK-3
```

```
                      Mature                                                Star
cgcauguugcucguuuaccuucuggaucuuguuugacagcacucccaacguuguuguuuggaugacauagaagugcugucgaacaugauccagaaggugcugucgaacaacauacaaaaaggggguuuacuuuacguaccgcccaucacgaucuucuucuucuucuucuuccugaaagaguaucaaacaugggguuucuguuuguuucucuuuuucuuuuuuaaaguagauuuacauguuaacuguauugc
......................uggaucuuguuGgacagcacuc........................................................................................................................................................................................   3       1    JCK-1
......................uggCucuuguuugacagcacuc........................................................................................................................................................................................   4       1    LT-2
......................uggaucuuguuugacagcacuA........................................................................................................................................................................................   38      1    JCK-1
......................uggaucuuguuugacaAcacuc........................................................................................................................................................................................   6       1    JCK-3
......................uggaucuuguuugCcagcacuc........................................................................................................................................................................................   19      1    JCK-3
......................uUgaucuuguuugacagcacuc........................................................................................................................................................................................   1       1    JCK-1
......................uggaucuuguuugacagcacuU........................................................................................................................................................................................   22      1    LCK-2
......................uggaucuuguuugCcagcacuc........................................................................................................................................................................................   44      1    JCK-2
......................ugAaucuuguuugacagcacuc........................................................................................................................................................................................   1       1    LT-2
......................uggaucuuguuugacagcacuU........................................................................................................................................................................................   68      1    JCK-1
......................uggaucuCguuugacagcacuc........................................................................................................................................................................................   2       1    JCK-1
......................uggaucuuguuugacagcacuc........................................................................................................................................................................................   220     0    JT-1
......................uUgaucuuguuugacagcacuc........................................................................................................................................................................................   1       1    LT-2
......................ugNaucuuguuugacagcacuc........................................................................................................................................................................................   2       1    LT-2
......................uggGucuuguuugacagcacuc........................................................................................................................................................................................   1       1    JCK-3
......................uggaucuuguuugacagcacuA........................................................................................................................................................................................   12      1    LCK-3
......................uggaucCuguuugacagcacuc........................................................................................................................................................................................   1       1    JCK-1
......................uggaucuuguuugacagcaNuc........................................................................................................................................................................................   11      1    JCK-1
......................uggaCcuuguuugacagcacuc........................................................................................................................................................................................   9       1    JCK-3
......................uggaucuuguuugNcagcacuc........................................................................................................................................................................................   1       1    LCK-1
......................uggaucuuguuugacagcacuc........................................................................................................................................................................................   9941    0    JCK-1
......................uggaucuugCuugacagcacuc........................................................................................................................................................................................   1       1    JCK-3
......................uNgaucuuguuugacagcacuc........................................................................................................................................................................................   17      1    JCK-2
......................uggaucuuguuugacagcacuA........................................................................................................................................................................................   32      1    LT-1
......................uggNucuuguuugacagcacuc........................................................................................................................................................................................   7       1    JCK-1
......................Cggaucuuguuugacagcacuc........................................................................................................................................................................................   5       1    JCK-3
......................uggaucuuAuuugacagcacuc........................................................................................................................................................................................   4       1    JCK-3
......................uggaucuuguuugacagcacuc........................................................................................................................................................................................   17179   0    JCK-2
......................uggaucuuguuugacCgcacuc........................................................................................................................................................................................   5       1    JCK-1
......................uggaucuuguuugacagcacuc........................................................................................................................................................................................   1069    0    LT-3
......................ugUaucuuguuugacagcacuc........................................................................................................................................................................................   1       1    JCK-1
......................uggaucuuguuugacagcacuc........................................................................................................................................................................................   1262    0    LT-1
......................uggaucuuguuugacagcacuG........................................................................................................................................................................................   2       1    JCK-3
......................uggaucuuguuugacCgcacuc........................................................................................................................................................................................   3       1    JCK-3
......................uggaucuuguuugacagcacGc........................................................................................................................................................................................   1       1    JCK-3
......................uggaCcuuguuugacagcacuc........................................................................................................................................................................................   1       1    LT-2
......................uggaucuuguuugacagcacuU........................................................................................................................................................................................   64      1    LCK-3
......................uggaucuuguuugacagcCcuc........................................................................................................................................................................................   1       1    JT-3
......................uggaucuuguuugacagcaUuc........................................................................................................................................................................................   8       1    JCK-3
......................uggaucuuguuugCcagcacuc........................................................................................................................................................................................   2       1    LT-2
......................ugAaucuuguuugacagcacuc........................................................................................................................................................................................   2       1    JCK-1
......................uggaucuuguuugacagcacuU........................................................................................................................................................................................   97      1    JCK-2
......................uggGucuuguuugacagcacuc........................................................................................................................................................................................   4       1    JCK-2
......................uggaucuuguuugacagcCcuc........................................................................................................................................................................................   1       1    LCK-1
......................uggCucuuguuugacagcacuc........................................................................................................................................................................................   1       1    JT-2
......................uUgaucuuguuugacagcacuc........................................................................................................................................................................................   1       1    JCK-2
......................uggaucuuAuuugacagcacuc........................................................................................................................................................................................   4       1    JCK-2
......................uggaucuuAuuugacagcacuc........................................................................................................................................................................................   1       1    LT-2
......................uggaucuuguuugacagcacuA........................................................................................................................................................................................   41      1    JCK-3
......................uggaucuuguuugaGagcacuc........................................................................................................................................................................................   4       1    JCK-2
......................uggaucuuguuugacagcCcuc........................................................................................................................................................................................   7       1    JCK-1
......................uggaucuuguuugacaAcacuc........................................................................................................................................................................................   3       1    JCK-2
......................uggaucuuguuuAacagcacuc........................................................................................................................................................................................   1       1    JCK-1
......................uggaucuuguuugacagNacuc........................................................................................................................................................................................   1       1    JCK-3
......................uAgaucuuguuugacagcacuc........................................................................................................................................................................................   3       1    JCK-1
......................uggaucuuguuugacagcacuU........................................................................................................................................................................................   48      1    LT-3
......................uggaucuuguuugacagcacuc........................................................................................................................................................................................   12872   0    JCK-3
......................uggaucuuguGugacagcacuc........................................................................................................................................................................................   5       1    JCK-1
......................uggaucuuguCugacagcacuc........................................................................................................................................................................................   1       1    JCK-1
......................ugAaucuuguuugacagcacuc........................................................................................................................................................................................   4       1    JCK-2
......................uAgaucuuguuugacagcacuc........................................................................................................................................................................................   10      1    JCK-3
......................uggaucuuNuuugacagcacuc........................................................................................................................................................................................   1       1    JCK-3
......................uggaucuuguuugacagcaAuc........................................................................................................................................................................................   1       1    JCK-2
......................uggaucuuguuNgacagcacuc........................................................................................................................................................................................   29      1    JCK-1
......................uggaucuuguuugacagcacuU........................................................................................................................................................................................   85      1    JCK-3
......................uggaucuuguuugacagcacuA........................................................................................................................................................................................   11      1    JT-1
......................uggaucuuguuugacagcacuU........................................................................................................................................................................................   18      1    LCK-1
......................uggaucuugCuugacagcacuc........................................................................................................................................................................................   1       1    JCK-2
......................uggaucuuguuugacagcUcuc........................................................................................................................................................................................   1       1    JCK-1
......................uggauUuuguuugacagcacuc........................................................................................................................................................................................   7       1    JCK-2
```

Mature Star

```
cgcauguugcucguuuaccuucuggaucuuguuugacagcacucccaacguuguuguuuggaugacauagaagugcugucgaacaugauccagaaggugcugucgaacaacauacaaaaaggguuuacuuuacguaccgcccaucacgaucuucuucuucuucuucuuccugaaagaguaucaaacaugggguuucuguuuguuucucuuuuucuuuuuuaaaguagauuuacauguuaacuguauugc
......................uggaucuuguuugacagcacuU.........................................................................................................................................................................................................   4   1   JT-3
......................uggaucuuguuugacagcacuA.........................................................................................................................................................................................................   59  1   JCK-2
......................uggaucuuguuugacagcacuc.........................................................................................................................................................................................................   70  0   LCK-2
......................uAgaucuuguuugacagcacuc.........................................................................................................................................................................................................   7   1   JCK-2
......................uNgaucuuguuugacagcacuc.........................................................................................................................................................................................................   15  1   JCK-3
......................uggaucuuguuugCcagcacuc.........................................................................................................................................................................................................   3   1   LCK-3
......................uggaucuuguuugacagcacuc.........................................................................................................................................................................................................   76  0   LCK-1
......................uggaucuugGuugacagcacuc.........................................................................................................................................................................................................   3   1   JCK-2
......................uggaGcuuguuugacagcacuc.........................................................................................................................................................................................................   1   1   JCK-3
......................uggCucuuguuugacagcacuc.........................................................................................................................................................................................................   36  1   JCK-1
......................uggaucuuguuuUacagcacuc.........................................................................................................................................................................................................   1   1   JCK-1
......................ugCaucuuguuugacagcacuc.........................................................................................................................................................................................................   1   1   JCK-2
......................uggCucuuguuugacagcacuc.........................................................................................................................................................................................................   1   1   JT-1
......................ugUaucuuguuugacagcacuc.........................................................................................................................................................................................................   3   1   JCK-3
......................uggaucuuguuugGcagcacuc.........................................................................................................................................................................................................   3   1   JCK-2
......................uggaucuuguuugacagcacuA.........................................................................................................................................................................................................   34  1   LT-3
......................uggaucuuguuugacagcacuG.........................................................................................................................................................................................................   4   1   JCK-2
......................uggaucuuguuugacagUacuc.........................................................................................................................................................................................................   1   1   JCK-1
......................uggauUuuguuugacagcacuc.........................................................................................................................................................................................................   5   1   JCK-1
......................uggaucuuguuugacagGacuc.........................................................................................................................................................................................................   7   1   JCK-1
......................uggaucuuguuugCcagcacuc.........................................................................................................................................................................................................   7   1   LT-1
......................uggaucuuguuugacagcNcuc.........................................................................................................................................................................................................   7   1   JCK-1
......................uggaucuuguuugacGgcacuc.........................................................................................................................................................................................................   1   1   LT-1
......................ugAaucuuguuugacagcacuc.........................................................................................................................................................................................................   2   1   LT-3
......................uggaucuuguuugacaAcacuc.........................................................................................................................................................................................................   4   1   JCK-1
......................uggaucuugGuugacagcacuc.........................................................................................................................................................................................................   3   1   JCK-1
......................Cggaucuuguuugacagcacuc.........................................................................................................................................................................................................   2   1   JCK-1
......................uggaucCuguuugacagcacuc.........................................................................................................................................................................................................   5   1   JCK-3
......................uggaucuuUuuugacagcacuc.........................................................................................................................................................................................................   1   1   JCK-3
......................uggauAuuguuugacagcacuc.........................................................................................................................................................................................................   1   1   JCK-1
......................uggaucuuguuugacagcaUuc.........................................................................................................................................................................................................   7   1   JCK-1
......................uggaucuuguuugacaCcacuc.........................................................................................................................................................................................................   1   1   JCK-3
......................uggaucuCguuugacagcacuc.........................................................................................................................................................................................................   3   1   JCK-2
......................uggaucuuguuugacagcacuN.........................................................................................................................................................................................................   4   1   LT-2
......................uggaucuuguuugacCgcacuc.........................................................................................................................................................................................................   2   1   LCK-3
......................uNgaucuuguuugacagcacuc.........................................................................................................................................................................................................   26  1   JCK-1
......................uggGucuuguuugacagcacuc.........................................................................................................................................................................................................   1   1   JCK-1
......................uggaucuuguuugacagcacuU.........................................................................................................................................................................................................   47  1   LT-1
......................uggaucuuguuugGcagcacuc.........................................................................................................................................................................................................   1   1   JCK-1
......................uggaucuuguuugacCgcacuc.........................................................................................................................................................................................................   1   1   LT-2
......................uggaucuuguuugacagcacNc.........................................................................................................................................................................................................   3   1   JCK-1
......................uggaucuuguuugacagcacuc.........................................................................................................................................................................................................   138 0   JT-2
......................uggaucuuguuugacagcacuc.........................................................................................................................................................................................................   627 0   LT-2
......................uggaucuuguuugacagcacGc.........................................................................................................................................................................................................   1   1   JCK-2
......................uggaucuuguuugacagcacuG.........................................................................................................................................................................................................   7   1   LT-1
......................uggaucuuguuugCcagcacuc.........................................................................................................................................................................................................   2   1   JT-1
......................uggaucuuguuugacagcaUuc.........................................................................................................................................................................................................   5   1   JCK-2
......................uggCucuuguuugacagcacuc.........................................................................................................................................................................................................   3   1   LCK-3
......................Cggaucuuguuugacagcacuc.........................................................................................................................................................................................................   1   1   LT-2
......................uggaucuuguuugacagcacuA.........................................................................................................................................................................................................   33  1   LT-2
......................uggaucuuguuugacagcGcuc.........................................................................................................................................................................................................   3   1   JCK-2
......................uggaucuuguuugacagcUcuc.........................................................................................................................................................................................................   2   1   JCK-2
......................uggaucuugGuugacagcacuc.........................................................................................................................................................................................................   3   1   JCK-3
......................uggaucuuguuugacagcacucG........................................................................................................................................................................................................   3   1   JCK-1
......................uggaucuuguuugacagcacuGc........................................................................................................................................................................................................   2   1   LT-2
......................uggaucuuguuugacagcacuUc........................................................................................................................................................................................................   6   1   LT-2
......................uggaucuuguuugacagcacucG........................................................................................................................................................................................................   4   1   JCK-3
......................uggaucuuguuugacagcacucc........................................................................................................................................................................................................   2   0   LT-2
......................uggaucuuguuugacagcacuGc........................................................................................................................................................................................................   17  1   LCK-3
......................uggaucuuguuugacagcacuUc........................................................................................................................................................................................................   5   1   LT-1
......................uggaucuuguuugacagcacucA........................................................................................................................................................................................................   24  1   LCK-3
......................uggaucuuguuugacagcacuUc........................................................................................................................................................................................................   5   1   LCK-3
......................uggaucuuguuugacagcacucA........................................................................................................................................................................................................   79  1   JCK-1
......................uggaucuuguuugacagcacucA........................................................................................................................................................................................................   10  1   JT-2
......................uggaucuuguuugacagcacucA........................................................................................................................................................................................................   23  1   LT-1
......................uggaucuuguuugacagcacuUc........................................................................................................................................................................................................   11  1   JCK-3
......................uggaucuuguuugacagcacuAc........................................................................................................................................................................................................   1   1   JCK-2
......................uggaucuuguuugacagcacuAc........................................................................................................................................................................................................   7   1   LCK-3
......................uggaucuuguuugacagcacuAc........................................................................................................................................................................................................   2   1   LT-2
......................uggaucuuguuugacagcacucG........................................................................................................................................................................................................   5   1   JCK-2
```

```
                      Mature                                             Star
cgcauguugcucguuuaccuucuggaucuuguuugacagcacucccaacguuguuguuuggaugacauagaagugcugucgaacaugauccagaaggugcugucgaacaacauacaaaaaggggguuuacuuuacguaccgcccaucacgaucuucuucuucuucuucuuccugaaagaguaucaaacaugggguuucuguuuguuucucuuuuucuuuuuuaaaguagauuuacauguuaacuguauugc
......................uggaucuuguuugacagcacuGc..............................................................................................................................................................................................................  1    1    LT-3
......................uggaucuuguuugacagcacucA..............................................................................................................................................................................................................  14   1    JT-3
......................uggaucuuguuugacagcacucA..............................................................................................................................................................................................................  14   1    LT-2
......................uggaucuuguuugacagcacuAc..............................................................................................................................................................................................................  6    1    LT-1
......................uggaucuuguuugacagcacucA..............................................................................................................................................................................................................  6    1    LT-3
......................uggaucuuguuugacagcacuUc..............................................................................................................................................................................................................  13   1    JCK-2
......................uggaucuuguuugacagcacuUc..............................................................................................................................................................................................................  5    1    JCK-1
......................uggaucuuguuugacagcacuGc..............................................................................................................................................................................................................  9    1    LT-1
......................uggaucuuguuugacagcacucA..............................................................................................................................................................................................................  100  1    JCK-3
......................uggaucuuguuugacagcacuUc..............................................................................................................................................................................................................  1    1    LT-3
......................uggaucuuguuugacagcacucA..............................................................................................................................................................................................................  140  1    JCK-2
......................uggaucuuguuugacagcacuccc.............................................................................................................................................................................................................  2    0    LT-3
......................uggaucuuguuugacagcacucAc.............................................................................................................................................................................................................  13   1    LCK-3
......................uggaucuuguuugacagcacucAc.............................................................................................................................................................................................................  2    1    LT-3
......................uggaucuuguuugacagcacucAc.............................................................................................................................................................................................................  7    1    LT-1
......................uggaucuuguuugacagcacuccU.............................................................................................................................................................................................................  1    1    LT-1
.......................ggaucuuguuugacagca..................................................................................................................................................................................................................  1    0    LT-1
.......................ggaucuuguuugacagca..................................................................................................................................................................................................................  2    0    JCK-3
.......................ggaucuuguuugacagca..................................................................................................................................................................................................................  1    0    JCK-1
.......................ggaucuuguuugacagcac.................................................................................................................................................................................................................  4    0    LCK-3
.......................ggaucuuguuugacagcac.................................................................................................................................................................................................................  2    0    LT-1
.......................ggaucuuguuugacagcac.................................................................................................................................................................................................................  1    0    LT-2
.......................ggaucuuguuugacagcacu................................................................................................................................................................................................................  5    0    LT-1
.......................ggaucuuguuugacagcacu................................................................................................................................................................................................................  2    0    LT-3
.......................ggaucuuguuugacagcacu................................................................................................................................................................................................................  5    0    JCK-1
.......................ggaucuuguuugacagcacu................................................................................................................................................................................................................  4    0    JCK-3
.......................ggaucuuguuugacagcacu................................................................................................................................................................................................................  5    0    LT-2
.......................ggaucuuguuugacagcacu................................................................................................................................................................................................................  2    0    LCK-3
.......................ggaucuuguuugacagcacu................................................................................................................................................................................................................  3    0    JCK-2
.......................ggaucuuguuugacagcacuc...............................................................................................................................................................................................................  14   0    JCK-3
.......................ggaucuuguuugacagcacuc...............................................................................................................................................................................................................  6    0    JCK-1
.......................ggaucuuguuugacagcacuc...............................................................................................................................................................................................................  6    0    JCK-2
.......................ggaucuuguuugacagcacucA..............................................................................................................................................................................................................  2    1    JCK-2
.......................ggaucuuguuugacagcacuccc.............................................................................................................................................................................................................  1    0    LT-1
........................gaucuuguuugacagcacu................................................................................................................................................................................................................  3    0    JCK-1
........................gaucuuguuugacagcacu................................................................................................................................................................................................................  3    0    LT-3
........................gaucuuguuugacagcacu................................................................................................................................................................................................................  14   0    LT-2
........................gaucuuguuugacagcacu................................................................................................................................................................................................................  1    0    JCK-2
........................gaucuuguuugacagcacu................................................................................................................................................................................................................  1    0    LT-1
........................gaucuuguuugacagcacuc...............................................................................................................................................................................................................  3    0    LT-3
........................gaucuuguuugacagcacuc...............................................................................................................................................................................................................  2    0    LT-1
........................gaucuuguuugacagcacucc..............................................................................................................................................................................................................  6    0    LT-2
........................gaucuuguuugacagcacucc..............................................................................................................................................................................................................  24   0    JCK-1
........................gaucuuguuugacagcacucc..............................................................................................................................................................................................................  8    0    LCK-3
........................gaucuuguuugacagcacucc..............................................................................................................................................................................................................  8    0    LT-1
........................gaucuuguuugacagcacucc..............................................................................................................................................................................................................  9    0    LT-3
........................gCucuuguuugacagcacucc..............................................................................................................................................................................................................  1    1    JCK-3
........................gaucuuguuugacagcacucc..............................................................................................................................................................................................................  26   0    JCK-3
........................gaucuuguuugCcagcacucc..............................................................................................................................................................................................................  1    1    LT-3
........................gaucuuguuugacagcacucc..............................................................................................................................................................................................................  32   0    JCK-2
........................gaucuuguuugacagcacucA..............................................................................................................................................................................................................  1    1    LT-1
.........................aucuuguuugacagcacu................................................................................................................................................................................................................  3    0    LT-2
.........................aucuuguuugacagcacu................................................................................................................................................................................................................  2    0    LT-1
.........................aucuuguuugacagcacuU...............................................................................................................................................................................................................  1    1    LT-2
.........................aucuuguuugacagcacuc...............................................................................................................................................................................................................  2    0    LT-1
.........................aucuuguuugacagcacuc...............................................................................................................................................................................................................  1    0    LT-2
.........................aucuuguuugacagcacuc...............................................................................................................................................................................................................  1    0    JCK-2
.........................aucuuguuugacagcacuc...............................................................................................................................................................................................................  5    0    LT-3
.........................aucuuguuugacagcacuc...............................................................................................................................................................................................................  8    0    LCK-3
.........................aucuuguuugacagcacucc..............................................................................................................................................................................................................  7    0    LT-2
.........................aucuuguuugacagcacucc..............................................................................................................................................................................................................  25   0    LCK-2
.........................aucuuguuugacagcacucc..............................................................................................................................................................................................................  23   0    LCK-1
.........................aucuuguuugacagcacucc..............................................................................................................................................................................................................  9    0    JCK-1
.........................aucuuguuugacagcacucc..............................................................................................................................................................................................................  4    0    LT-1
.........................aucuuguuugacagcacucc..............................................................................................................................................................................................................  12   0    LT-3
.........................aucuuguuugacagcacucc..............................................................................................................................................................................................................  11   0    LCK-3
.........................aucuuguuugacagcacucA..............................................................................................................................................................................................................  2    1    LT-1
.........................aucuuguuugacagcacucc..............................................................................................................................................................................................................  17   0    JCK-3
.........................aucuuguuugacCgcacucc..............................................................................................................................................................................................................  1    1    JCK-3
.........................aucuuguuugacagcacucc..............................................................................................................................................................................................................  12   0    JCK-2
```

Mature Star

```
cgcauguugcucguuuaccuucuggaucuuguuugacagcacucccaacguuguuguuuggaugacauagaagugcugucgaacaugauccagaaggugcugucgaacaacauacaaaaaggggguuuacuuuacguaccgcccaucacgaucuucuucuucuucuucuuccugaaagaguaucaaacaugggguuucuguuuguuucucuuuuucuuuuuuaaaguagauuuacauguuaacuguauugc
.........................aucuuguuugacagcacucA..............................................................................................................................................................................................   1   1   LT-2
.........................aucuuguuugacagcCcucc..............................................................................................................................................................................................   1   1   LT-2
.........................aucuuguuugacagcacucA..............................................................................................................................................................................................   7   1   LCK-3
.........................aucuuguuugacCgcacuccc.............................................................................................................................................................................................   1   1   LT-2
.........................aucuuguuugacagcacuccc.............................................................................................................................................................................................   39  0   LT-1
.........................aucuuguuugacagcacuccc.............................................................................................................................................................................................   58  0   JCK-3
.........................Cucuuguuugacagcacuccc.............................................................................................................................................................................................   1   1   LCK-2
.........................aucuuguuugacagcacuccA.............................................................................................................................................................................................   1   1   LT-3
.........................aucuuguuugacagcacuccc.............................................................................................................................................................................................   85  0   JCK-2
.........................aucuuguuugacagcacuccc.............................................................................................................................................................................................   12  0   LCK-1
.........................aucuuguuugacCgcacuccc.............................................................................................................................................................................................   1   1   JCK-3
.........................Cucuuguuugacagcacuccc.............................................................................................................................................................................................   1   1   JCK-1
.........................aucuuguuugacagcacuccc.............................................................................................................................................................................................   5   0   LCK-2
.........................aucuuguuugacagcacuccc.............................................................................................................................................................................................   52  0   JCK-1
.........................aucuuguuugacagcacuccc.............................................................................................................................................................................................   17  0   JT-1
.........................aucuuguuugacagcacuccc.............................................................................................................................................................................................   58  0   LT-2
.........................aucuuguuugaUagcacuccc.............................................................................................................................................................................................   1   1   JCK-2
.........................aucuuguuugacagcacuccc.............................................................................................................................................................................................   50  0   LT-3
.........................aucuuguuugacagcacuccc.............................................................................................................................................................................................   32  0   LCK-3
.........................aucuuguuugacCgcacuccc.............................................................................................................................................................................................   1   1   JCK-2
.........................aucuuguuugacagcacucccU............................................................................................................................................................................................   1   1   JCK-1
.........................aucuuguuugacagcacucccU............................................................................................................................................................................................   1   1   LT-3
.........................aucuuguuugacagcacucccU............................................................................................................................................................................................   1   1   JCK-2
.........................aucuuguuugacagcacuccca............................................................................................................................................................................................   2   0   JCK-2
.........................aucuuguuugacagcacucccU............................................................................................................................................................................................   1   1   JCK-3
..........................ucuuguuugacagcacuc...............................................................................................................................................................................................   2   0   LT-3
..........................ucuuguuugacagcacuc...............................................................................................................................................................................................   1   0   LT-2
..........................ucuuguuugacagcaUucc..............................................................................................................................................................................................   1   1   JCK-2
..........................ucuuguuugacagcaUucc..............................................................................................................................................................................................   1   1   JCK-1
..........................ucuuguuugacagcacucA..............................................................................................................................................................................................   1   1   LT-3
..........................ucuuguuugacagcacucc..............................................................................................................................................................................................   2   0   LT-3
..........................ucuuguuugacagcacucc..............................................................................................................................................................................................   3   0   LT-2
..........................ucuuguuugacagcacucc..............................................................................................................................................................................................   1   0   LT-1
..........................ucuuguuugacagcacuccU.............................................................................................................................................................................................   1   1   JCK-2
..........................ucuuguuugacagcacuccc.............................................................................................................................................................................................   14  0   LT-1
..........................ucuuguuugacagcacuccc.............................................................................................................................................................................................   7   0   JCK-3
..........................ucuuguuugacagcacuccU.............................................................................................................................................................................................   1   1   LT-3
..........................ucuuguuugacagcacuccc.............................................................................................................................................................................................   6   0   JCK-1
..........................ucuuguuugacagcacuccc.............................................................................................................................................................................................   10  0   JCK-2
..........................ucuuguuugacagcacuccc.............................................................................................................................................................................................   2   0   LT-2
..........................ucuuguuugacagcacuccc.............................................................................................................................................................................................   18  0   LT-3
..........................ucuuguuugacagcacuccc.............................................................................................................................................................................................   5   0   LCK-3
..........................ucuuguuugacagcacuccca............................................................................................................................................................................................   1   0   JCK-2
..........................ucuuguuugacagcacuccca............................................................................................................................................................................................   1   0   LT-1
..........................ucuuguuugacagcacuccca............................................................................................................................................................................................   3   0   LT-3
..........................ucuuguuugacagcacuccca............................................................................................................................................................................................   1   0   LT-2
..........................ucuuguuugacagcacuccca............................................................................................................................................................................................   1   0   JCK-3
..........................ucuuguuugacagcacucccaacg.........................................................................................................................................................................................   1   0   JCK-1
..........................ucuuguuugacagcacucccaacg.........................................................................................................................................................................................   3   0   LCK-3
..........................ucuuguuugacagcacucccaacg.........................................................................................................................................................................................   2   0   LT-2
..........................ucuuguuugacagcacucccaacg.........................................................................................................................................................................................   1   0   JCK-2
............................uuguuCgacagcacucccaacg.........................................................................................................................................................................................   1   1   JCK-3
............................uuguuugacagcacucccaacguug......................................................................................................................................................................................   2   0   LT-2
...............................uuugacagcacucccaac..........................................................................................................................................................................................   1   0   LT-3
...............................uuugacagcacucccaac..........................................................................................................................................................................................   1   0   LT-1
...............................uuugacagcacucccaacg.........................................................................................................................................................................................   2   0   LT-3
...............................uuugacagcacucccaacg.........................................................................................................................................................................................   1   0   JCK-1
...............................uuugacagcacucccaacg.........................................................................................................................................................................................   4   0   LCK-3
...............................uuugacagcacucccaacgu........................................................................................................................................................................................   1   0   LT-2
...............................uuCgacagcacucccaacgu........................................................................................................................................................................................   2   1   JCK-3
...............................uuugacagcacucccaacguu.......................................................................................................................................................................................   1   0   LT-3
...............................uuCgacagcacucccaacguu.......................................................................................................................................................................................   1   1   JCK-2
...............................uuugacagcacucccaacguug......................................................................................................................................................................................   1   0   JCK-2
...............................uuugacagcacucccaacguug......................................................................................................................................................................................   1   0   JCK-3
...........................................cccaacguuguuguuuggaugacauagaag..................................................................................................................................................................................   1   0   JCK-1
...........................................cccaacguuguuguuuggaugacauagaag..................................................................................................................................................................................   1   0   LT-2
...........................................cccaacguuguuguuuggaugacauagaag..................................................................................................................................................................................   2   0   JCK-2
...........................................cccaacguuguuguuuggaugacauagaag..................................................................................................................................................................................   4   0   JCK-3
...................................................uguuguuuggaugacauagaagugc...............................................................................................................................................................................   2   0   JCK-1
.....................................................uuguuuggaugacauagaagug................................................................................................................................................................................   1   0   LT-3
```

Mature Star

cgcauguugcucguuuaccuucuggaucuuguuugacagcacucccaacguuguuguuuggaugacauagaagugcugucgaacaugauccagaaggugcugucgaacaacauacaaaaaggguuuacuuuacguaccgcccaucacgaucuucuucuucuucuucuuccugaaagaguaucaaacaugggguuucuguuuguuucucuuuucuuuuuuaaaguagauuuacauguuaacuguauugc

uuguuuggaugacauagaagugcug	2	0	LCK-3
uuguuuggaugacauagaagugcug	2	0	LT-1
uuguuuggaugacauagaagugcugucg	1	0	LT-2
uuguuuggaugacauagaagugcugucgaa	1	0	LT-2
uguuuggaugacauagaag	2	0	LT-1
uguuuggaugacauagaagugcuguc	6	0	JCK-1
uuuggaugacauagaagugc	1	0	LT-1
uuuggaugacauagaagugcuguc	1	0	LT-2
uuuggaugacauagaagugcugucgaac	2	0	LT-1
uuuggaugacauagaagugcugucgaac	1	0	LT-2
uuuggaugacauagaagugcugucgaacC	1	1	LT-1
uuggaugacauagaagug	2	0	LT-3
uuggaugacauagaagugcugucga	2	0	LT-1
uuggaugacauagaagugcugucgaacU	1	1	LT-3
uuggaugacauagaagugcugucgaaca	1	0	JCK-2
uggaugacauagaagugcugucgaa	4	0	LCK-3
ggaugacauagaagugcugucgaaca	3	0	JCK-3
ggaugacauagaagugcugucgaaca	1	0	JCK-2
gaugacauagaagugcugu	1	0	LT-3
augacauagaagugcugucgaacU	1	1	JCK-1
ugacauagaagugcugucgaa	1	0	LT-2
acauagaagugcugucgaac	3	0	LT-2
acauagaagugcugucgaac	3	0	JCK-1
acauagaagugcugucgaac	2	0	JCK-3
acauagaagugcugucgaac	3	0	JCK-2
acauagaagugcugucgaaca	1	0	LT-3
acauagaagugcugucgaaca	14	0	LCK-3
acauagaagugcugucgGacaug	9	1	JT-2
acauagaagugcugucgGacaug	6	1	JT-3
acauagaagugcugucgaacaug	1	0	LT-1
acauagaagugcugucgaacauga	1	0	LT-3
cauagaagugcugucgaaA	4	1	JT-2
cauagaagugcugucgaaA	2	1	JT-3
cauagaagugcugucgaacauU	6	1	LCK-3
auagaagugcugucgaac	1	0	LT-3
auagaagugcugucgaacU	1	1	JCK-3
auagaagugcugucgaacaugauc	1	0	LT-1
auagaagugcugucgaacaugauc	1	0	JCK-1
auagaagugcugucgaacaugauc	1	0	LT-2
auagaagugcugucgaacaugauc	2	0	JCK-2
auagaagugcugucgaacaugauc	1	0	JCK-3
auagaagugcugucgGacaugauc	1	1	JCK-2
auagaagugcugucgaacaugaucA	1	1	LT-1
auagaagugcugucgaacaugauccaga	1	0	JCK-3
auagaagugcugucgaacaugauccaga	1	0	JCK-2
uagaagugcugucgGacaugauc	1	1	JCK-2
gaagugcugucgaacauga	2	0	LT-2
gaagugcugucgGacaugauc	1	1	JCK-1
gaagugcugucgGacaugauc	1	1	JCK-2
gaagugcugucgaacaugaucca	2	0	LT-2
aagugcugucgaacauga	17	0	LCK-2
aagugcugucgaacauga	6	0	JT-3
aagugcugucgaacauga	17	0	LCK-1
aagugcugucgaacauga	12	0	JT-1
aagugcugucgaacauga	1	0	JCK-2
aagugcugucgaacauga	2	0	LT-3
aagugcugucgaacauga	5	0	JT-2
aagugcugucgaacauAa	2	1	LCK-3
aagugcugucgaacauga	2	0	JCK-3
aagugcugucgaacauga	5	0	LCK-3
aagugcugucgaacaugau	1	0	LT-2
aagugcugucgaacaugUu	1	1	JCK-1
aagugcugucgGacaugau	2	1	JCK-2
aagugcugucgaacaugau	1	0	LT-3
aagugcugucgaacaugaA	1	1	LT-3
aagugcugucgaacaugUu	1	1	JCK-3
aagugcugucgaacaugUu	3	1	LCK-3
aagugcugucgGacaugau	1	1	JCK-3
aagugcugucgaacaugau	4	0	LT-1
aagugcugucgGacaugau	1	1	JCK-1

Mature Star

cgcauguugcucguuuaccuucuggaucuuguuugacagcacucccaacguuguuguuuggaugacauagaagugcugucgaacaugauccagaaggugcugucgaacaacauacaaaaggguuuacuuuacguaccgcccaucacgaucuucuucuucuucuucuuccugaaagaguaucaaacaugggguuucuguuuguuucucuuuuucuuuuuuaaaguagauuuacauguuaacuguauugc

aagugcugucgaacaugUu	6	1	LT-2
aagugcugucgaacaugau	6	0	LCK-3
aagugcugucgGacaugauc	37	1	JT-1
aagugcugucgaacaugauc	7	0	JT-2
aagugcugucgaacaugauc	11	0	LCK-3
aagugcugucgaCcaugauc	1	1	LT-3
aagugcugucgaacaugauc	17	0	JCK-3
aagugcugucgaacaugauc	19	0	LT-2
aagugcugucgaacaugauc	19	0	JT-3
aagugcugucgaacauAauc	1	1	JCK-3
aagugcugucgaacaugauc	26	0	JCK-2
aagugcugucgaacaugauc	15	0	JCK-1
aagugcugucgaacaugauN	1	1	JCK-1
aagugcugucgGacaugauc	119	1	JCK-2
aagugcugucgaacaugauc	24	0	LT-1
aagugcugucgaacaugauU	1	1	LT-1
aagugcugucgGacaugauc	73	1	JCK-1
aagugcugucgaacauAauc	1	1	JCK-2
aagugcugucgGacaugauc	84	1	JCK-3
aagugcugucgaacaugauc	24	0	LT-3
aagugcugucgaacaugauU	5	1	LT-2
aagugcugucgUacaugaucc	1	1	JCK-3
aagugcugucgaacaugaucU	2	1	JCK-3
aagugcugucgaacaugaucc	74	0	JCK-2
aagugcugucgGacaugaucc	137	1	JCK-1
aagugcugucgaacaugaucA	1	1	LT-3
aagugcugucgaacaugaucc	1	0	JT-3
aagugcugucgaaNaugaucc	1	1	LCK-1
aagugcugucgaacaugaucc	64	0	JCK-3
Cagugcugucgaacaugaucc	1	1	LT-1
aagugcugucgaacaugaucc	5	0	JT-2
aagugcugucgaacaugaucc	57	0	LT-1
aagugcugucgGacaugaucc	248	1	JCK-2
aagugcugucgaacaugaucc	22	0	LCK-1
aagugcugucgaacaugaucc	64	0	JT-1
aagugcugucgGacaugaucc	17	1	JT-3
aagugcugucgaacaugCucc	1	1	JCK-1
aagugcugucgGacaugaucc	8	1	JT-2
aagugcugucgaacaugaucc	63	0	LCK-3
aCgugcugucgaacaugaucc	4	1	LT-2
aagugcugucgaacaugaucc	57	0	JCK-1
Cagugcugucgaacaugaucc	1	1	JCK-2
aagugcugucgaacaugaucc	26	0	LCK-2
aagugcugucgGacaugaucc	191	1	JCK-3
Cagugcugucgaacaugaucc	1	1	LCK-3
aagugcugucgaacaugaucc	106	0	LT-3
aagugcugucgGacaugaucc	13	1	JT-1
aagugcugucgaacNugaucc	1	1	LCK-1
aagugcugucgaacaugaucU	1	1	JCK-2
aagGgcugucgaacaugaucc	1	1	JCK-1
aagugcugucgaacaugaucc	94	0	LT-2
aagugcugucgaacaugaucca	1	0	LT-3
aagugcugucgaacaugaucca	4	0	LT-2
Cagugcugucgaacaugaucca	1	1	JCK-3
aagugcugucgaacaugauccaA	3	1	LT-1
aagugcugucgaacaugauccaA	3	1	JCK-1
aagugcugucgaacaugauccag	5	0	LT-1
aagugcugucgGacaugauccag	3	1	JCK-1
aagugcugucgaacaugauccag	2	0	LT-2
aagugcugucgaacaugauccag	7	0	JCK-3
aagugcugucgaacaugauccag	8	0	JCK-2
aagugcugucgaacaugauccag	2	0	LT-3
aagugcugucgGacaugauccag	4	1	JCK-2
aagugcugucgGacaugauccag	2	1	JCK-3
aagugcugucgaacaugauccag	4	0	JCK-1
aagugcugucgaaAaugauccaga	1	1	LT-2
aCgugcugucgaacaugauccaga	1	1	JCK-3
aagugcugucgaacauUauccaga	1	1	JCK-2
aagugcugucgGacaugauccaga	27	1	JCK-1
aagugcugucgaacaugauccagU	2	1	JCK-2

Mature **Star**

```
cgcauguugcucguuuaccuucuggaucuuguuugacagcacucccaacguuguuguuuggaugacauagaagugcugucgaacaugauccagaaggugcugucgaacaacauacaaaaagggguuuacuuuacguaccgcccaucacgaucuucuucuucuucuucuuccugaaagaguaucaaacaugggguuucuguuuguuucucuuuuucuuuuuuaaaguagauuuacauguuaacuguauugc
......................................................................aagugcugucgaacaugauccagU............................................................................................................................................................	1	1	JCK-3
......................................................................aagugcugucgaacaugauccaga............................................................................................................................................................	71	0	JCK-2
......................................................................aagugcugucgaCcaugauccaga............................................................................................................................................................	1	1	LT-2
......................................................................aagugcugucgaacaugauccaga............................................................................................................................................................	42	0	JCK-3
......................................................................aagugcugucgaacaugauccagU............................................................................................................................................................	2	1	JCK-1
......................................................................aagugcugucgGacaugauccaga............................................................................................................................................................	39	1	JCK-3
......................................................................aagugcugucgGacaugauccaga............................................................................................................................................................	48	1	JCK-2
......................................................................aagugcugucgaacaugauccaga............................................................................................................................................................	26	0	LT-1
......................................................................aagugcugucgaacaugauccaga............................................................................................................................................................	15	0	LT-2
......................................................................aNgugcugucgaacaugauccaga............................................................................................................................................................	1	1	JCK-3
......................................................................aagugcugucgaacaugauccaga............................................................................................................................................................	34	0	JCK-1
......................................................................aagugcugucgaacaugauccaga............................................................................................................................................................	13	0	LCK-3
......................................................................aagugcugucgaacaugauccaga............................................................................................................................................................	7	0	JT-1
......................................................................aagugcugucgaacaugauccaga............................................................................................................................................................	21	0	LT-3
......................................................................aagugcugucgaacaugauAcaga............................................................................................................................................................	1	1	JCK-1
.......................................................................agugcugucgaacaugUu.................................................................................................................................................................	9	1	LT-3
.......................................................................agugcugucgaacaugUu.................................................................................................................................................................	1	1	JCK-3
.......................................................................agugcugucgaacaugUu.................................................................................................................................................................	8	1	LT-2
.......................................................................agugcugucgaacauAau.................................................................................................................................................................	7	1	LCK-3
.......................................................................agugcugucgaacaugau.................................................................................................................................................................	16	0	LCK-2
.......................................................................agugcugucgaacaugau.................................................................................................................................................................	19	0	LT-2
.......................................................................agugcugucgaacaugau.................................................................................................................................................................	1	0	JCK-2
.......................................................................agugcugucgaacaugau.................................................................................................................................................................	5	0	LT-3
.......................................................................agugcugucgaacaugau.................................................................................................................................................................	4	0	LT-1
.......................................................................agugcugucgaacaugaC.................................................................................................................................................................	1	1	JCK-3
.......................................................................agugcugucgaacaugau.................................................................................................................................................................	17	0	LCK-1
.......................................................................agugcugucgaacaugUu.................................................................................................................................................................	22	1	LCK-3
.......................................................................agugcugucgCacaugau.................................................................................................................................................................	1	1	LCK-2
.......................................................................agugcugucgaacaugUu.................................................................................................................................................................	1	1	LT-1
.......................................................................agugcugucgaacGugau.................................................................................................................................................................	1	1	LCK-1
.......................................................................agugcugucgaacaugauc................................................................................................................................................................	42	0	LT-1
.......................................................................agugcugucgaacaugauU................................................................................................................................................................	4	1	LT-1
.......................................................................agugcugucgaacaugauc................................................................................................................................................................	1	0	JT-3
.......................................................................agugcugucgaNcaugauc................................................................................................................................................................	1	1	JCK-1
.......................................................................agugcugucgaacCugauc................................................................................................................................................................	1	1	LCK-1
.......................................................................agugcugucgaacaugauU................................................................................................................................................................	3	1	JCK-1
.......................................................................agugcugucgaacaugauc................................................................................................................................................................	69	0	LT-2
.......................................................................agugcugucgaacaugauA................................................................................................................................................................	1	1	LT-1
.......................................................................agugcugucgGacaugauc................................................................................................................................................................	3	1	JCK-3
.......................................................................agugcugucgaacaugauc................................................................................................................................................................	94	0	JCK-2
.......................................................................agugcugucgaacaugauc................................................................................................................................................................	17	0	LCK-3
.......................................................................agugcugucgaacaugauc................................................................................................................................................................	24	0	LCK-2
.......................................................................agugcugucgaacaugauU................................................................................................................................................................	7	1	JCK-2
.......................................................................agugcugucgaacaugauU................................................................................................................................................................	16	1	LT-3
.......................................................................agugcugucgaacaugauc................................................................................................................................................................	68	0	JCK-3
.......................................................................agugcugucgaacaugauU................................................................................................................................................................	15	1	LCK-3
.......................................................................agugcugucgGacaugauc................................................................................................................................................................	5	1	JCK-1
.......................................................................agugcugucgaacaugauc................................................................................................................................................................	63	0	JCK-1
.......................................................................agugcugucgaacaugauc................................................................................................................................................................	15	0	LCK-1
.......................................................................agNgcugucgaacaugauc................................................................................................................................................................	1	1	LT-2
.......................................................................agugcugucgaacaugauc................................................................................................................................................................	88	0	LT-3
.......................................................................agugcugucgGacaugauc................................................................................................................................................................	10	1	JCK-2
.......................................................................agugcugucgaacaugauU................................................................................................................................................................	13	1	LT-2
.......................................................................agugcugucgaacaugaucU...............................................................................................................................................................	4	1	LT-3
.......................................................................agugcugucgaacaugaucc...............................................................................................................................................................	26	0	JCK-1
.......................................................................agugcugucgaacaugaucc...............................................................................................................................................................	22	0	LCK-1
.......................................................................agugcugucgaacaugaucA...............................................................................................................................................................	1	1	JCK-3
.......................................................................agugcugucgaacaugauUc...............................................................................................................................................................	1	1	LT-3
.......................................................................agugcugucgaacaugaucc...............................................................................................................................................................	72	0	LT-1
.......................................................................agugcugucgaacaugaucc...............................................................................................................................................................	38	0	JCK-3
.......................................................................agugcugucgaacaugauUc...............................................................................................................................................................	1	1	LT-1
.......................................................................agugcuguAgaacaugaucc...............................................................................................................................................................	1	1	JCK-2
.......................................................................agugcugucgGacaugaucc...............................................................................................................................................................	7	1	JCK-1
.......................................................................agugcugucgaacCugaucc...............................................................................................................................................................	1	1	LT-2
.......................................................................aguAcugucgaacaugaucc...............................................................................................................................................................	1	1	LT-2
.......................................................................agugcugucgaacaugaucA...............................................................................................................................................................	1	1	LT-3
.......................................................................agugcugucgaacaugaucc...............................................................................................................................................................	90	0	LCK-3
.......................................................................agugcugucgaCcaugaucc...............................................................................................................................................................	1	1	LT-3
.......................................................................agugcugucgGacaugaucc...............................................................................................................................................................	3	1	JCK-3
.......................................................................agugcugucgaacaugaucA...............................................................................................................................................................	4	1	JT-3
```

```
                      Mature                                             Star
cgcauguugcucguuuaccuucuggaucuuguuugacagcacucccaacguuguuguuuggaugacauagaagugcugucgaacaugauccagaaggugcugucgaacaacauacaaaaagggguuuacuuuacguaccgcccaucacgaucuucuucuucuucuucuuccugaaagaguaucaaacaugggguuucuguuuguuucucuuuuucuuuuuuaaaguagauuuacauguuaacuguauugc
.......................................................................agugcugucgaacaugaucc...............................................................................................................................................................  49    0  JCK-2
.......................................................................agugcugucgaacaugaucc...............................................................................................................................................................  118   0  LT-2
.......................................................................agugcugucgaacaugaucc...............................................................................................................................................................  130   0  LT-3
.......................................................................agugcugucgGacaugaucc...............................................................................................................................................................  9     1  JCK-2
.......................................................................agugcugucgaacaugaucc...............................................................................................................................................................  21    0  LCK-2
.......................................................................agugcugucgaacaugaucU...............................................................................................................................................................  2     1  LT-2
.......................................................................agugcugucgaacaugaucc...............................................................................................................................................................  5     0  JT-2
.......................................................................agugcugucgaacaugaucc...............................................................................................................................................................  8     0  JT-3
.......................................................................Cgugcugucgaacaugaucc...............................................................................................................................................................  1     1  LT-1
.......................................................................aAugcugucgaacaugaucc...............................................................................................................................................................  1     1  LT-2
.......................................................................agugcugucgaacaugaucA...............................................................................................................................................................  4     1  JT-2
.......................................................................agugcugucgaacaugCucc...............................................................................................................................................................  2     1  LT-2
.......................................................................agugcugucgaacaugaucU...............................................................................................................................................................  2     1  LT-1
.......................................................................agugcugucgaCcaugaucca..............................................................................................................................................................  1     1  JCK-1
.......................................................................agugcugucgaacaugauccU..............................................................................................................................................................  1     1  LT-1
.......................................................................agugcugucgaacaugauccC..............................................................................................................................................................  3     1  LT-3
.......................................................................agugcugucgaacaugauccC..............................................................................................................................................................  6     1  JCK-2
.......................................................................agugcugucgaacaugauccU..............................................................................................................................................................  3     1  JCK-3
.......................................................................agugcugucgaacaugaucca..............................................................................................................................................................  152   0  JCK-1
.......................................................................agugcugucgaacaugauccC..............................................................................................................................................................  1     1  LT-1
.......................................................................agugcugucgGacaugaucca..............................................................................................................................................................  10    1  JCK-3
.......................................................................agugcugucgGacaugaucca..............................................................................................................................................................  19    1  JCK-2
.......................................................................agugcugucgaacaugaucca..............................................................................................................................................................  53    0  LCK-2
.......................................................................agugcugucgaacaugaucca..............................................................................................................................................................  93    0  LT-2
.......................................................................agugcugucgaacaugauccC..............................................................................................................................................................  1     1  JCK-1
.......................................................................agugcugucgaacaugauccC..............................................................................................................................................................  2     1  JCK-3
.......................................................................agugcugucgaacaugauccU..............................................................................................................................................................  2     1  JCK-1
.......................................................................agugcugucgaacaugaucca..............................................................................................................................................................  121   0  LT-3
.......................................................................agugcugucgaacaugaucca..............................................................................................................................................................  226   0  JCK-2
.......................................................................agugcugucgaacaugaucca..............................................................................................................................................................  43    0  LCK-1
.......................................................................agugcugucgaacaGgaucca..............................................................................................................................................................  1     1  LCK-1
.......................................................................agugcugucgaacaugaucNa..............................................................................................................................................................  1     1  JCK-1
.......................................................................agugcugucgaacaugauccU..............................................................................................................................................................  1     1  JCK-2
.......................................................................aNugcugucgaacaugaucca..............................................................................................................................................................  1     1  JCK-3
.......................................................................agugcugucgaacaugauccU..............................................................................................................................................................  2     1  LT-2
.......................................................................agugcugucgaCcaugaucca..............................................................................................................................................................  1     1  LT-2
.......................................................................agugcugucgaacaugCucca..............................................................................................................................................................  1     1  LCK-3
.......................................................................agugcugucgaacaugaucca..............................................................................................................................................................  115   0  LT-1
.......................................................................agugcugucgaacaugaucca..............................................................................................................................................................  24    0  JT-1
.......................................................................agugcugucgaacaugaucca..............................................................................................................................................................  144   0  JCK-3
.......................................................................agugcugucgGacaugaucca..............................................................................................................................................................  13    1  JCK-1
.......................................................................agugcugucgaacaugaucca..............................................................................................................................................................  36    0  LCK-3
.......................................................................agugcugucgaacaugauccag.............................................................................................................................................................  2     0  JCK-1
.......................................................................agugcugucgGacaugauccaga............................................................................................................................................................  2     1  JCK-3
.......................................................................agugcugucgaacaugauccaga............................................................................................................................................................  3     0  JCK-1
.......................................................................agugcugucgGacaugauccaga............................................................................................................................................................  3     1  JCK-2
.......................................................................agugcugucgaacaugauccaga............................................................................................................................................................  2     0  JCK-3
.......................................................................agugcugucgaacaugauccaga............................................................................................................................................................  2     0  JCK-2
.......................................................................agugcugucgGacaugauccaga............................................................................................................................................................  1     1  JCK-1
........................................................................gugcugucgaacaugauA................................................................................................................................................................  1     1  LT-3
........................................................................gugcugucgaacaugauA................................................................................................................................................................  1     1  LT-1
........................................................................gugcugucgGacaugauc................................................................................................................................................................  5     1  JCK-3
........................................................................gugcugucgGacaugauc................................................................................................................................................................  3     1  JCK-1
........................................................................gugcugucgGacaugauc................................................................................................................................................................  6     1  JCK-2
........................................................................gugcugucgaCcaugaucc...............................................................................................................................................................  1     1  LT-2
........................................................................gugcugucgaacaugauUc...............................................................................................................................................................  1     1  LT-1
........................................................................gugcugucgaacaugauccU..............................................................................................................................................................  1     1  LT-2
........................................................................gugcugucgaacaugaucca..............................................................................................................................................................  12    0  LT-1
........................................................................gugcugucgaacaugaucca..............................................................................................................................................................  18    0  LT-2
........................................................................gugcugucgaacaugaucca..............................................................................................................................................................  9     0  LCK-3
........................................................................gugcugucgGacaugaucca..............................................................................................................................................................  1     1  JCK-2
........................................................................gugcugucgaacaugaucca..............................................................................................................................................................  16    0  LT-3
........................................................................gugcugucgaGcaugaucca..............................................................................................................................................................  1     1  LT-3
........................................................................gugcugucgaacaugaucca..............................................................................................................................................................  14    0  JCK-2
........................................................................gugcugucgaacaugaucca..............................................................................................................................................................  7     0  JCK-1
........................................................................gugcugucgaacaugaucca..............................................................................................................................................................  4     0  JCK-3
........................................................................gugcugucgaacaugauccaA.............................................................................................................................................................  1     1  JCK-2
........................................................................gugcugucgaacaugauccag.............................................................................................................................................................  17    0  LT-1
........................................................................gugcugucgaacaugauccag.............................................................................................................................................................  15    0  JCK-2
........................................................................gugcugucgaacaugauccag.............................................................................................................................................................  9     0  JCK-1
```

Mature **Star**

cgcauguugcucguuuaccuucuggaucuuguuugacagcacucccaacguuguuguuuggaugacauagaagugcugucgaacaugauccagaaggugcugucgaacaacauacaaaaaggggguuuacuuuacguaccgcccaucacgaucuucuucuucuucuucuuccugaaagaguaucaaacauggggguuucuguuuguuucucuuuuucuuuuuuaaaguagauuuacauguuaacuguauugc			
gugcugucgaacaugauccag	14	0	LT-3
gugcugucgaacaugauccag	24	0	JCK-3
gugcugucgGacaugauccag	2	1	JCK-3
gugcugucgaacaugauccag	10	0	LT-2
Cugcugucgaacaugauccag	1	1	LT-3
gugcugucgaacaugauccag	6	0	LCK-3
gugcugucgGacaugauccag	2	1	JCK-1
gugcugucgGacaugauccag	5	1	JCK-2
Cugcugucgaacaugauccaga	1	1	JCK-3
Cugcugucgaacaugauccaga	2	1	JCK-2
gugcugucgaacaugauccagU	1	1	LCK-3
gugcugucgaacaugauccagU	1	1	LT-3
gugcugucgaacaugauccaga	1	0	JCK-2
gugcugucgGacaugauccaga	1	1	JCK-2
gugcugucgaacaugauccagU	2	1	JCK-1
gugcugucgaacaugauccaga	2	0	JCK-3
gugcugucgaacaugauccagU	1	1	LT-1
Cugcugucgaacaugauccaga	2	1	JCK-1
Cugcugucgaacaugauccaga	2	1	LT-3
gugcugucgGacaugauccaga	3	1	JCK-1
gugcugucgaacaugauccaga	1	0	JCK-1
ugcugucgaacaugaucU	31	1	LCK-1
ugcugucgaacaugaucU	1	1	JCK-2
ugcugucgaacaugaucU	1	1	LT-1
ugcugucgGacaugaucc	28	1	JT-1
ugcugucgGacaugaucc	140	1	JCK-3
ugcugucgaacaugaucU	16	1	LCK-3
ugcugucgGacaugaucc	5	1	JT-2
ugcugucgGacaugaucc	112	1	JCK-1
ugcugucgaacCugaucc	1	1	LCK-3
ugcugucgGacaugaucc	3	1	JT-3
ugcugucgaacaugaucU	2	1	LT-3
ugcugucgGacaugaucc	195	1	JCK-2
ugcugucgaacaugaucU	19	1	LCK-2
ugcugucgaacaugaucA	4	1	LT-1
ugcugucgaacaugaucA	1	1	LT-3
ugcugucgaacaugaucU	2	1	LT-2
ugcugucgGacaugaucca	7	1	JT-1
ugcugucgGacaugaucca	41	1	JCK-1
ugcugucgaacaugaucca	125	0	LT-2
ugcugucgaacaugauccU	1	1	LT-1
ugcugucgaaAaugaucca	1	1	LT-2
ugcugucgGacaugaucca	3	1	JT-3
ugcugucgGacaugaucca	59	1	JCK-2
ugcugucgaacaugaucca	18	0	JCK-3
ugcugucgaacaugaucca	67	0	JT-1
uAcugucgaacaugaucca	1	1	LCK-3
ugcugucgaacaugaucca	24	0	JCK-1
ugcugucgaacaugCucca	1	1	LT-2
ugcugucgaacaugaucca	53	0	LCK-1
ugcugucgaacaugaucca	6	0	JT-2
ugcugucgaacGugaucca	1	1	LCK-3
ugcugucgaacaugaCcca	1	1	LCK-3
ugcugucgaacaugaucca	81	0	LCK-3
ugcugucgaacaugaucca	61	0	LT-1
ugcugucgaacaugaucca	26	0	JCK-2
ugcugucgaacaugaucca	4	0	JT-3
ugcugucgGacaugaucca	30	1	JCK-3
ugcugucgaacaugaucca	50	0	LCK-2
ugcugucgaacaugaucca	96	0	LT-3
ugcugucgaacaugauccaA	2	1	LT-3
ugcugucgaacaugauccag	117	0	LCK-2
ugcugucgaacaugauccaA	2	1	JCK-2
ugcugucgGacaugauccag	497	1	JCK-3
ugcugucgCacaugauccag	1	1	JCK-3
ugcugucgGacaugauccag	29	1	JT-2
ugcugucgaacaugauccaC	1	1	LT-3
ugcugucgaacaugauccag	262	0	JCK-2
ugcugucgaCcaugauccag	1	1	LT-3
ugNugucgaacaugauccag	1	1	LT-2

Mature **Star**

cgcauguugcucguuuaccuucuggaucuuguuugacagcacucccaacguuguuguuuggaugacauagaagugcugucgaacaugauccagaaggugcugucgaacaacauacaaaaaggggguuuacuuuacguaccgcccaucacgaucuucuucuucuucuucuuccugaaagaguaucaaacaugggguuucuguuuguuucucuuuuucuuuuuuaaaguagauuuacauguuaacuguauugc

ugcNgucgaacaugauccag	1	1	JCK-1
ugcugCcgaacaugauccag	1	1	LT-3
ugcugucgGacaugauccag	431	1	JCK-1
ugcugucgaacaugauccag	458	0	LT-2
ugcugucgaacaugCuccag	1	1	LT-2
ugcugucAaacaugauccag	1	1	LT-2
ugcugucgaacaugauccag	441	0	LT-1
ugcugucgaacaugauccag	143	0	JCK-1
uNcugucgaacaugauccag	1	1	JT-1
ugcugucgaacaugauccag	73	0	JT-3
ugcugucgaacaugCuccag	1	1	LT-3
ugcugucgaacaugauccCg	1	1	LT-3
ugcugucgaacCugauccag	2	1	LT-1
ugcugucgaacaugCuccag	1	1	LT-1
ugcugucgaacCugauccag	1	1	LT-2
ugcugucgCacaugauccag	1	1	LT-1
ugcugucgaacCugauccag	1	1	LCK-2
ugcugucgaacaugauccag	530	0	LT-3
ugcugucgaacaugGuccag	1	1	JT-1
ugcugucgaacaugauccag	114	0	LCK-1
ugcugucgGacaugauccag	49	1	JT-1
ugcugucgaacaugauccaN	1	1	JCK-1
ugcugAcgaacaugauccag	1	1	LT-3
ugcugucgGacaugauccag	64	1	JT-3
ugcugucgaacaugauccaU	1	1	JCK-1
uAcugucgaacaugauccag	1	1	LT-2
ugcugucgaacaugauccag	45	0	JT-2
ugcugucgaacaugauccag	169	0	JCK-3
ugcugucgaCcaugauccag	1	1	LT-2
uNcugucgaacaugauccag	1	1	JCK-3
ugcugucgaCcaugauccag	1	1	LCK-2
ugcugucgaacaugauccag	40	0	JT-1
ugcugucgGacaugauccag	787	1	JCK-2
ugcugucgaacaugauccag	487	0	LCK-3
ugcugucgCacaugauccag	2	1	LT-2
ugcugucgCacaugauccag	1	1	LCK-3
ugcugucgaacaugauccaA	2	1	JCK-3
ugcugucgaCcaugauccag	1	1	LCK-3
ugcugucgaacaugCuccaga	4	1	JCK-3
ugcugucgaCcaugauccaga	3	1	JCK-1
ugcugucgCacaugauccaga	3	1	JCK-1
ugcugucgaacaugaucUaga	1	1	LT-3
ugcugucgaacaugauccaAa	5	1	JCK-3
ugcugucgaacaugauccaga	125	0	LCK-1
ugcugucgGacaugauccaga	44	1	JT-1
ugcugucgaacCugauccaga	2	1	LT-2
ugcugucgaacaugauccaga	2163	0	JCK-3
ugcugucgaacGugauccaga	1	1	JCK-2
ugcugucgaacaugauccagU	2	1	JCK-2
uNcugucgaacaugauccaga	4	1	JCK-2
ugcugucgaacaugCuccaga	7	1	LT-2
uAcugucgaacaugauccaga	1	1	LCK-3
ugcugucgaacaugauccaga	1673	0	JCK-1
ugcugucgGacaugauccaga	20	1	JT-3
ugcugucgNacaugauccaga	2	1	JCK-3
ugcuguAgaacaugauccaga	1	1	JCK-2
ugcugucgaacaugauccCga	4	1	JCK-2
ugUugucgaacaugauccaga	1	1	LT-3
ugcugucgaacaugauccagC	1	1	JT-1
ugcugucgaacaugCuccaga	3	1	LT-3
uAcugucgaacaugauccaga	1	1	JCK-3
Cgcugucgaacaugauccaga	1	1	LT-1
ugcugucgaacCugauccaga	5	1	JCK-2
ugcugucgaacaugauGcaga	2	1	JCK-1
ugcugucgCacaugauccaga	5	1	JCK-2
ugGugucgaacaugauccaga	1	1	LT-2
ugcuAucgaacaugauccaga	1	1	LT-3
ugcugucgaCcaugauccaga	3	1	JCK-2
ugcugucgaacaugauccCga	5	1	LT-2
ugcugucgaacaugauccagC	1	1	JCK-1

Mature **Star**

cgcauguugcucguuuaccuucuggaucuuguuugacagcacucccaacguuguuguuuggaugacauagaagugcugucgaacaugauccagaaggugcugucgaacaacauacaaaaaggguuuacuuuacguaccgcccaucacgaucuucuucuucuucuucuuccugaaagaguaucaaacaugggguuucuguuuguuucucuuuuucuuuuuaaaguagauuuacauguuaacuguauugc

ugcugucgaacaugauccGga	1	1	JCK-3
uUcugucgaacaugauccaga	2	1	LT-2
ugcugucgaacGugauccaga	1	1	LCK-3
ugcugucgCacaugauccaga	2	1	LCK-3
ugcugucgaacaugaucAaga	1	1	JCK-2
ugcugucgCacaugauccaga	10	1	LT-2
uAcugucgaacaugauccaga	1	1	LT-2
ugcugucgaacCugauccaga	1	1	JCK-1
ugcuguAgaacaugauccaga	1	1	LT-2
ugcugucgaacaugauccaga	186	0	JT-1
ugcCgucgaacaugauccaga	2	1	LT-1
ugcugucgaacaugaucNaga	1	1	JCK-3
ugcugucgaacGugauccaga	1	1	JCK-3
ugcugucgaacaugauccaga	2228	0	LT-2
ugcugucgCacaugauccaga	1	1	LT-1
ugcugucgaCcaugauccaga	1	1	JCK-3
ugcugucAaacaugauccaga	1	1	JCK-2
ugcugucgaacaugaucAaga	3	1	LT-3
ugcuguUgaacaugauccaga	1	1	JCK-2
ugcuguUgaacaugauccaga	1	1	LT-1
ugcugucgaacNugauccaga	4	1	JCK-1
ugcugucUaacaugauccaga	1	1	LT-1
ugcugCcgaacaugauccaga	1	1	JCK-2
ugcugucgaacaugauccaga	2141	0	LT-1
ugcugucgaacauAauccaga	1	1	JCK-2
uAcugucgaacaugauccaga	1	1	JCK-1
ugcugucgaacaugauccCga	1	1	JCK-3
ugcugucgGacaugauccaga	7517	1	JCK-1
ugcugucgaacauAauccaga	3	1	LT-2
uAcugucgaacaugauccaga	4	1	LT-3
ugcugucgaacaugGuccaga	1	1	JCK-3
ugcugucgaacaugauccaAa	1	1	JCK-2
ugNugucgaacaugauccaga	7	1	LT-2
uAcugucgaacaugauccaga	1	1	JCK-2
ugcNgucgaacaugauccaga	4	1	JCK-1
ugcugCcgaacaugauccaga	2	1	LT-3
ugcugucgaacauAauccaga	2	1	JCK-3
ugcugucgCacaugauccaga	3	1	LT-3
ugcugucAaacaugauccaga	2	1	LT-2
ugcugCcgaacaugauccaga	1	1	JCK-3
ugcuguAgaacaugauccaga	1	1	LT-1
ugcugucgaacaugauccaga	118	0	JT-2
ugcugucgaacaugauccaga	2567	0	LT-3
ugcugucgCacaugauccaga	3	1	JCK-3
uUcugucgaacaugauccaga	1	1	JCK-2
ugcuguUgaacaugauccaga	1	1	JCK-3
uNcugucgaacaugauccaga	3	1	JCK-1
ugcugucgaacaugCuccaga	1	1	JCK-1
ugcugucgaacaugauccaNa	4	1	JCK-1
ugcugucgaacaugCuccaga	3	1	JCK-2
ugcugucgGacaugauccaga	17	1	JT-2
ugcugucgaCcaugauccaga	5	1	LT-3
ugcugucgaacaugauccagU	5	1	JCK-1
ugcugucgGacaugauccaga	9455	1	JCK-3
ugcugucgaacauAauccaga	2	1	LT-1
ugcugucgaacaugauccagC	1	1	JT-3
ugcugucAaacaugauccaga	1	1	LT-1
ugcugucgaacaugauccaga	1025	0	LCK-3
ugcugucgaacCugauccaga	1	1	LT-1
ugcugucgaCcaugauccaga	5	1	LT-2
ugcugucgaGcaugauccaga	1	1	JCK-3
uCcugucgaacaugauccaga	1	1	LT-2
uAcugucgaacaugauccaga	3	1	LT-1
ugcugucgaCcaugauccaga	1	1	LCK-3
ugcugucgaacaugauccagC	1	1	LT-3
ugcugucgaacaugauccaga	107	0	LCK-2
ugcugucgaGcaugauccaga	1	1	JCK-2
ugAugucgaacaugauccaga	1	1	LCK-3
uNcugucgaacaugauccaga	3	1	JCK-3
ugcugucgaacaugauccaga	145	0	JT-3

Mature **Star**

cgcauguugcucguuuaccuuc**uggaucuuguuugacagcacu**cccaacguuguuguuuggaugacauagaag**ugcugucgaacaugauccaga**aggugcugucgaacaacauacaaaaaggggguuuacuuuacguaccgcccaucacgaucuucuucuucuucuucuuccugaaagaguaucaaacaugggguuucuguuuguuucucuuuuucuuuuuuaaaguagauuuacauguuaacuguauugc

ugcugucgaacaCgauccaga	1	1	LT-1
ugcugucgaacaugauccagC	1	1	JCK-2
ugcugucgaCcaugauccaga	3	1	LT-1
ugcugucgGacaugauccaga	12782	1	JCK-2
ugcugucgaacCugauccaga	2	1	LT-3
ugcugucgaacaugaCccaga	1	1	LT-3
ugcugucgaacaugauccCga	5	1	LT-3
ugcugucgaacaugauccaga	2920	0	JCK-2
ugcugucgUacaugauccaga	1	1	JCK-2
uUcugucgaacaugauccaga	1	1	JCK-1
ugcugucgaaAaugauccaga	1	1	LT-1
ugcugucgaacaugauccagaC	2	1	LT-3
ugcugucgaacaugauccagaC	3	1	JCK-2
ugcugucgaCcaugauccagaa	1	1	LT-1
ugcugucgaacaugauccagaU	3	1	JCK-3
ugcugucgaacaugauccagaC	3	1	LT-1
ugcugucgaacaugauccagaU	4	1	LT-3
ugcugucgaacaugauccagaU	3	1	LT-2
ugcugucgaacaugauccagaa	1	0	JCK-1
ugcugucgaacaugauccagaC	12	1	LCK-3
ugcugucgGacaugauccagaa	12	1	JCK-2
ugcugucgaacaugauccagaa	3	0	LT-2
ugcugucgaacaugauccagaa	2	0	JCK-2
ugcugucgGacaugauccagaa	14	1	JCK-3
ugcugucgaacaugauccagaa	3	0	LT-3
ugcugucgaacaugauccagaC	1	1	JCK-3
ugcugucgaacaugauccagaU	1	1	LT-1
ugcugucgaacaugauccagaC	8	1	LT-2
ugcugucgaacaugauccagaa	6	0	LT-1
ugcugucgaacaugauccagaU	1	1	JCK-2
ugcugucgGacaugauccagaa	5	1	JCK-1
ugcugucgaacaugauccagaagg	2	0	JCK-2
ugcugucgaacaugauccagaagg	1	0	JCK-3
gcugucgaacaugauccag	6	0	LCK-3
gcugucgGacaugauccaga	5	1	JCK-3
gcugucgGacaugauccaga	9	1	JCK-2
gcugucgGacaugauccaga	2	1	JCK-1
cugucgaacaugauccag	1	0	LT-3
cugucgaacaugauccaga	7	0	LT-1
cugucgaacaugauccaga	21	0	LT-2
cugucgaacCugauccaga	1	1	LT-2
cugucgaacaugauccaga	8	0	LCK-3
cugucgaacaugauccagU	1	1	LT-2
cugucgaacaugauccaga	10	0	LT-3
cugucgCacaugauccaga	1	1	LT-1
cugucgaacaugauccaga	3	0	JCK-2
cugucgaacaugauccagaU	1	1	LT-1
cugucgaacaugauccagaa	4	0	LT-1
cugucgaacaugauccagaa	1	0	JCK-3
cugucgaacaugauccagUa	2	1	LT-2
cugucgaacaugauccagaa	1	0	LT-2
cugucgaacaugauccagaa	3	0	LT-3
cugucgaacaugauccagaa	2	0	JCK-2
cugucgaacaugauccagaa	4	0	LCK-3
cugucgaacaugauccagaU	7	1	LCK-3
cugucgaacaugauccagaag	13	0	JCK-2
cugucgaacaugauccagaaU	3	1	LT-1
cugucgaacaAgauccagaag	9	1	LCK-3
cugucgaacaugauccagaag	18	0	JCK-3
cugucgaacaugauccagaag	21	0	LCK-3
cugucgaacaugauccagaaA	1	1	JCK-2
cugucgaacaugauccagaag	12	0	LT-1
cugucgaacaugauccagaag	20	0	LT-3
cugucgCacaugauccagaag	1	1	LT-1
cugucgaacaugauccagaag	10	0	LT-2
cugucgaacaugauccagaag	9	0	JCK-1
cugucgCacaugauccagaag	1	1	LT-2
cugucgaacaugauccagaagA	3	1	JCK-2
cugucgaacaugauccagaagUu	2	1	LT-2
cugucgaacaugauccagaagUu	1	1	LT-3

Mature **Star**

cgcauguugcucguuuaccuucuggaucuuguuugacagcacucccaacguuguuguuuggaugacauagaagugcugucgaacaugauccagaaggugcugucgaacaacauacaaaaggggguuuacuuuacguaccgcccaucacgaucuucuucuucuucuucuuccugaaagaguaucaaacaugggguuucuguuuguuucucuuuucuuuuuuaaaguagauuuacauguuaacuguauugc

ugucgaacaugauccaga	2	0	LT-1
ugucgaacaugauccaga	1	0	LT-3
ugucgaacaugauccagaagg	2	0	JCK-3
ugucgaacaugauccagaagg	5	0	LT-1
ugucgaacaugauccagaagg	4	0	LT-3
ugucgaacaugauccagaagg	6	0	LT-2
ugucgaacaugauccagaagg	2	0	JCK-2
ucgaacaugauccagaag	1	0	LT-1
ucgaacaugauccagaagg	1	0	JCK-1
ucgaacaugauccagaaggu	1	0	LT-2
ucgaacaugauccagaaggug	9	0	LT-2
ucgaacaugauccagaaggug	2	0	JCK-2
ucgaacaugauccagaagguA	1	1	LT-1
ucgaacaugauccagaaggug	16	0	LT-1
ucgaacaugauccagaaggug	23	0	LCK-2
ucgaacaugauccagaaggug	1	0	JCK-1
ucgaacaugauccagaaggug	27	0	LCK-1
ucgaacaugauccagaaggug	15	0	LT-3
cgaacaugauccagaaggu	2	0	LT-3
cgaacaugauccagaaggu	1	0	LT-2
cgaacCugauccagaaggu	1	1	LT-3
cgaacaugauccagaaggug	1	0	JCK-2
cgaacaugauccagaaggug	2	0	LT-3
cgaacaugauccagaaggug	3	0	LT-1
cgaacaugauccagaaggugc	6	0	LT-1
cgaacaugauccagaaggugc	3	0	LT-3
cgaacaugauccagaaggugc	4	0	JCK-3
cgaacaugauccagaaggugc	3	0	JCK-1
cgaacaugauccagaaggugc	6	0	JCK-2
aacaugauccagaaggugcugucgaaca	2	0	JCK-3
acaugauccagaaggugcugucg	1	0	LT-3
acaAgauccagaaggugcugucga	2	1	JCK-3
acaAgauccagaaggugcugucga	2	1	JCK-1
acaAgauccagaaggugcugucga	1	1	JCK-2
acaugauccagaaggugcugucgaa	1	0	JCK-1
acaugauccagaaggugcugucgaac	2	0	JCK-1
acaugauccagaaggugcugucgaac	1	0	JCK-3
acaugauccagaaggugcugucgaac	1	0	JCK-2
caugauccagaaggugcugucgaaca	1	0	LT-1
uccagaaggugcugucgaaca	2	0	LT-2
uccagaaggugcugucgaaca	2	0	LT-1
uccagaaggugcugucgaaca	1	0	LT-3
uccagaaggugcugucgaacaa	3	0	LT-1
uccagaaggugcugucgaacaa	1	0	LT-3
uccagaaggugcugucgaacaac	15	0	JCK-3
uccagaaggugcugucgaacaac	5	0	LCK-3
uccagaaggugcugucgaacaac	8	0	LT-3
uccagaaggugcugucgaacaac	12	0	JCK-2
uccagaaggugcugucgaacaac	4	0	LT-2
uccagaaggugcugucgaacaac	2	0	LT-1
uccagaaggugcugucgaacaac	4	0	JCK-1
uccagaaggugcugucgaacaacU	2	1	LT-1
uccagaaggugcugucgaacaacU	1	1	LT-3
uccagaaggugcugucgaacaaca	2	0	LT-2
uccagaaggugcugucgaacaacU	6	1	LCK-3
uccagaaggugcugucgaacaaca	3	0	LT-3
uccagaaggugcugucgaacaacN	1	1	LT-2
uccagaaggugcugucgaacaaca	2	0	JCK-3
uccagaaggugcugucgaacaacU	1	1	JCK-2
agaaggugcugucgaaca	1	0	LT-3
agaaggugcugucgaacaaca	4	0	LCK-3
agaUggugcugucgaacaaca	1	1	JCK-3
agaUggugcugucgaacaaca	1	1	JCK-2
agaaggugcugucgaacaacau	1	0	LT-1
agaaggugcugucgaacaacau	1	0	LT-3
agaaggugcugucgaacaacaua	2	0	LT-1
agaaggugcugucgaacaacaua	2	0	LT-2
gaaggugcugucgaacaacauacaaaaag	1	0	JCK-1
gugcugucgaacaaGaua	1	1	JCK-2
gugcugucgaacaaGaua	2	1	LCK-3

```
                     Mature                                         Star
cgcauguugcucguuuaccuucuggaucuuguuugacagcacucccaacguuguuguuuggaugacauagaagugcugucgaacaugauccagaaggugcugucgaacaacauacaaaagggguuuacuuuacguaccgcccaucacgaucuucuucuucuucuucuuccugaaagaguaucaaacaugggguuucuguuuguuucucuuuuucuuuuuuaaaguagauuuacauguuaacuguauugc
......................................................................................................................................................................uuccugaaagaguaucaaac...............................................................   5    0    LT-2
................................................................................................................................................................................................uuucuguuuguAucucuuuu.....................................   15   1    JT-1
................................................................................................................................................................................................uuucuguuuguAucucuuuu.....................................   1    1    LT-1
................................................................................................................................................................................................uuucuguuuguAucucuuuu.....................................   2    1    LCK-3
................................................................................................................................................................................................uuucuguuuguAucucuuuuuc...................................   1    1    LT-1
................................................................................................................................................................................................uuucuguuuguAucucuuuuuc...................................   1    1    JCK-3
................................................................................................................................................................................................uuucuguuuguAucucuuuuuc...................................   6    1    JT-1
................................................................................................................................................................................................uuucuguuuguAucucuuuuuc...................................   2    1    JCK-2
.................................................................................................................................................................................................uucuguuuguAucucuuuu.....................................   1    1    LT-2
.................................................................................................................................................................................................uucuguuuguAucucuuuuuc...................................   4    1    LT-1
.................................................................................................................................................................................................uucuguuuguAucucuuuuuc...................................   3    1    LT-3
..................................................................................................................................................................................................ucuguuuguAucucuuuuuc...................................   7    1    LCK-3
..................................................................................................................................................................................................ucuguuuguAucucuuuuuc...................................   1    1    LT-3
...................................................................................................................................................................................................cuguuuguAucucuuuuuc...................................   4    1    LT-1
...................................................................................................................................................................................................cuguuuguAucucuuuuuc...................................   1    1    LT-3
....................................................................................................................................................................................................uguuuguAucucuuuuuc...................................   2    1    LT-3
```
